# PRMT1 in Health and Disease: Emerging Perspectives From Molecular Mechanisms to Therapeutic Strategies

**DOI:** 10.1002/mco2.70482

**Published:** 2025-11-16

**Authors:** Yanqun Luo, Ying Gao, Xiaoliang Deng, Lei Wang, Tao Wu

**Affiliations:** ^1^ Department of Hepatology Longhua Hospital Shanghai University of Traditional Chinese Medicine Shanghai China; ^2^ Institute of Interdisciplinary Integrative Medicine Research Shanghai University of Traditional Chinese Medicine Shanghai China

**Keywords:** cancer, chronic liver diseases, cardiovascular diseases, inhibitor, protein arginine methyltransferase 1

## Abstract

Protein arginine methyltransferase 1 (PRMT1) serves as a critical epigenetic modulator involved in a wide range of physiological and pathological processes. Previous studies have established its fundamental roles in essential cellular mechanisms such as DNA repair, transcriptional regulation, and signal transduction. Dysregulation of PRMT1 has been further associated with the pathogenesis of various diseases, including cancer, metabolic disorders, and immune dysfunction. However, a systematic synthesis of the multifaceted functions of PRMT1 across these diverse pathological contexts remains lacking. This review seeks to address this gap by comprehensively examining the molecular mechanisms, biological functions, and context‐dependent roles of PRMT1. We integrate recent advances spanning multiple disease domains, with a particular focus on cancer, chronic liver diseases, cardiovascular disorders, neurodegenerative conditions, and immune‐related pathologies. In addition, we elucidate the mechanistic links between PRMT1 dysregulation and disease pathogenesis. Further, the development and clinical potential of small‐molecule inhibitors are also summarized. This review offers new perspectives on PRMT1‐related disease mechanisms and lays a theoretical foundation for the development of targeted therapies. Ultimately, this review aims to contribute to the progression of precision medicine and the enhancement of global health outcomes.

## Graphical abstract

Protein arginine methyltransferase 1 (PRMT1) is a pivotal enzyme that catalyzes the asymmetric dimethylation of arginine residues, thereby functioning as a critical epigenetic regulator of diverse biological processes such as gene expression, RNA splicing, and DNA repair. Consequently, PRMT1 dysregulation is implicated in the pathogenesis of numerous human diseases, including cancer, cardiovascular disorders, and neurodegenerative and immunological conditions. In oncology, aberrant PRMT1 activity drives key malignant phenotypes such as tumor proliferation, metastasis, therapeutic resistance, and immune evasion. Beyond cancer, its dysfunction also contributes to pathological processes such as hepatic lipid dysmetabolism, adverse cardiac remodeling, and neuronal damage. This central role in disease pathogenesis has motivated the development of small‐molecule PRMT1 inhibitors, with compounds such as MS023 and CTS‐2190 demonstrating promising efficacy in preclinical models. In this review, we comprehensively summarize the molecular and physiological functions of PRMT1, delineate its multifaceted roles in various diseases, and critically evaluate its potential as a therapeutic target alongside the associated challenges.

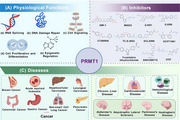

## 1 Introduction

Over the past few decades, posttranslational modifications (PTMs) have emerged as a major research focus in biomedical sciences due to their crucial role in regulating protein function. They are pivotal modulators of protein stability, activity, localization, and protein–protein interactions [1]. Among the various PTMs, arginine methylation has garnered significant attention. This modification is catalyzed by the protein arginine methyltransferase (PRMT) family, which adds one or two methyl groups to the arginine residues of proteins [2]. Given their regulatory significance, PRMT family members, including PRMT1, have become pivotal subjects for elucidating the mechanisms of disease pathogenesis. Among these, PRMT1 is regarded as one of the most critical due to its central role in a diverse array of cellular and physiological processes [3].

PRMT1, a principal mammalian type I PRMT, was first identified in 1996. It is predominantly found in large molecular complexes of 300–400 kDa and catalyzes the monomethylation and asymmetric dimethylation of arginine residues on protein substrates [3, 4]. Although initially characterized as a histone methyltransferase, PRMT1 is now known to methylate a broad range of both histone and nonhistone proteins. It is well known for methylating histone H4 on arginine 3 (H4R3me2a) [5]. Owing to its diverse substrate profile, PRMT1 is a critical regulator of numerous biological processes, including signal transduction, DNA repair, and transcriptional control [6]. The regulatory mechanisms controlling PRMT1's methyltransferase activity are complex and have been the subject of extensive research. PRMT1 was initially discovered through its interaction with members of the BTG/Tob family, B‐cell translocation gene 1 (BTG1) and BTG2, which are implicated in the suppression of cell proliferation. Specifically, BTG2 has been established as a direct substrate of PRMT1, and the coexistence of BTG1 and BTG2 enhances the methyltransferase activity of BTG2 [7]. The mammalian PRMT family comprises nine members, which are classified into three types based on their catalytic products: type I PRMTs (PRMT1, PRMT2, PRMT3, PRMT4/CARM1, PRMT6, and PRMT8) predominantly catalyze the synthesis of asymmetric dimethylarginine (ADMA) and monomethylated arginine (MMA). The primary functions of type II PRMTs (PRMT5 and PRMT9) involve the production of MMA and symmetric dimethylarginine. The principal catalyst for MMA production is type III PRMT (PRMT7) [8].

The precise regulation of PRMT1 activity is crucial for cellular homeostasis, and its dysregulation is strongly implicated in the pathogenesis of numerous human diseases. Under physiological conditions, PRMT1 catalyzes the H4R3me2a as a mark of transcriptional activation, thereby synergizing with other transcription factors to initiate the expression of downstream genes [9]. Moreover, PRMT1 is capable of methylating a wide range of nonhistone substrates, including p53, nuclear factor kappa B (NF‐κB), MRE11, and hnRNP family proteins. Through these modifications, PRMT1 precisely modulates their functions, thereby ensuring appropriate cellular responses to alterations in both the intracellular and extracellular environments [10]. Nevertheless, in various malignant tumors, the aberrant overexpression or heightened activity of PRMT1 is a frequently observed feature [11, 12, 13, 14, 15]. In these malignancies, PRMT1 functions as a pivotal oncogene by several methods, such as facilitating cell proliferation, suppressing apoptosis, augmenting cell motility and invasion, and influencing the tumor microenvironment. Beyond its pivotal function in oncology, the dysregulation of PRMT1 is also intricately associated with cardiovascular disorders. The principal catalytic product of PRMT1, ADMA, serves as a competitive inhibitor of endogenous nitric oxide (NO) synthase (NOS). Increased ADMA levels can result in endothelial dysfunction, irregular vascular tone, and atherosclerosis, thereby establishing PRMT1 as a significant target in cardiovascular disease (CVD) research [16]. Additionally, emerging research has begun to elucidate the potential role of PRMT1 in chronic liver diseases (CLDs), such as nonalcoholic fatty liver disease (NAFLD) [17], neurodegenerative diseases (NDs) including Parkinson's disease (PD) [18], and immune‐related diseases [19]. Moreover, as our understanding of the roles of PRMT1 in various diseases deepens, its potential as a therapeutic target has become increasingly evident. Consequently, the development of highly selective and potent small‐molecule PRMT1 inhibitors has advanced considerably, with several candidates, such as CTS‐2190, progressing to clinical trials, thereby presenting promising avenues for novel therapeutics [20].

While the significance of PRMT1 in health and disease is increasingly recognized, the field lacks a singular, integrative review of its molecular mechanisms, pathophysiology, and therapeutic potential. This review aims to address this critical gap by systematically elucidating the multifaceted functions of PRMT1. We first dissect its molecular architecture and fundamental biological roles. The core of this review is dedicated to examining the mechanistic underpinnings of PRMT1 dysregulation in oncogenesis, metabolic disorders, and immune dysfunction. Furthermore, we critically evaluate current therapeutic interventions, with a focus on small‐molecule inhibitors, and discuss the principal challenges and future directions for their clinical translation. Ultimately, this review provides a robust conceptual framework to guide future investigations into PRMT1‐driven pathologies and accelerate the development of precision therapeutics.

## 2 | Structures of PRMT1

### 2.1 Genomic Organization

**FIGURE 1:**
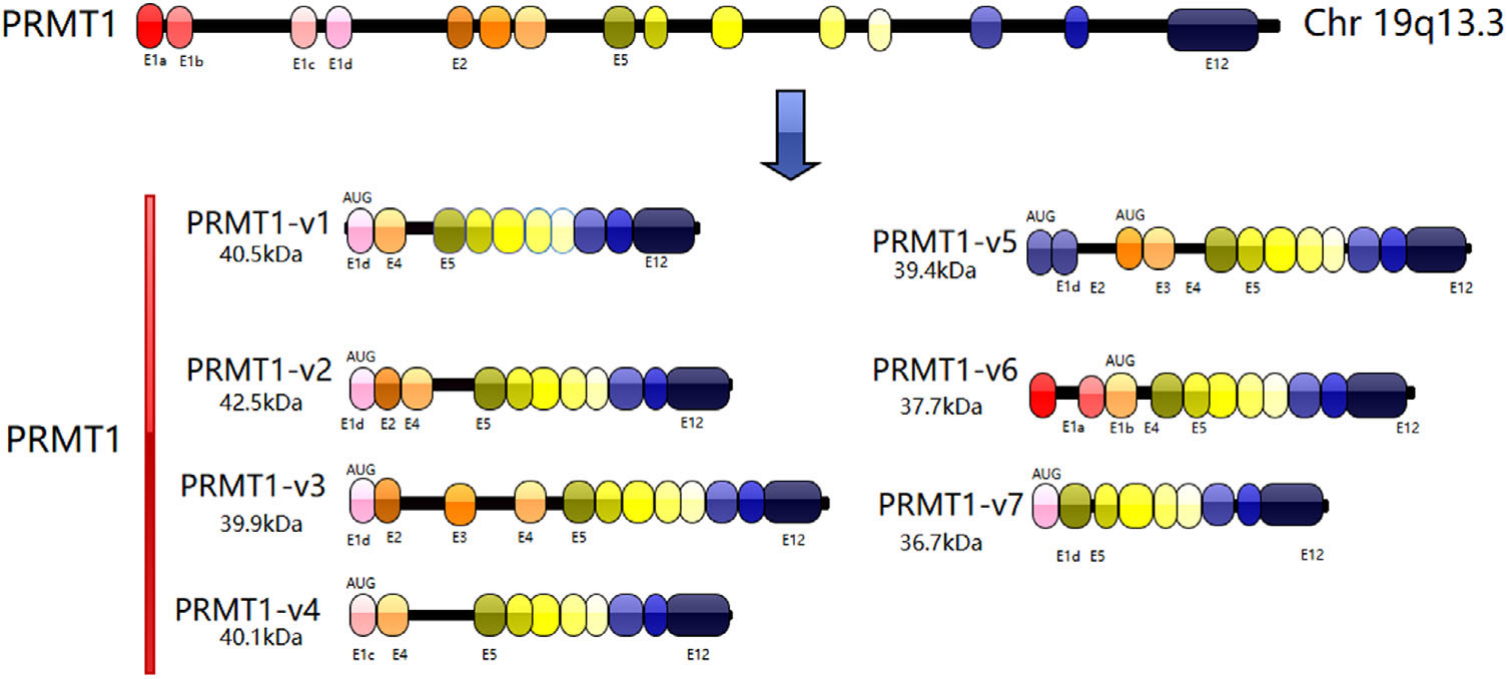
The genomic structure of PRMT1. The PRMT1 gene features a genomic architecture including 12 constitutive exons, with the first exon split into four alternative exons. This gene undergoes transcription and subsequent splicing, resulting in the creation of seven different PRMT1 isoforms, labeled v1 to v7. The distinct exon makeup differs among various isoforms, illustrating the gene's potential for alternative splicing. (adapted from Ref. [3]).

PRMT1 is situated on human chromosome 19q13.3, comprising 12 exons and 11 introns, with a total length of 11.2 kb and encoding 324 amino acids [21]. The genes for interferon regulatory factor 3 and associated RAS viral oncogene homologs are next to PRMT1. It is interesting to note that its transcription is opposite that of R‐RAS and IRF3. It catalyzes the synthesis of ω‐NG‐MMA and ω‐NG, NG‐ADMA and contributes to over 90% of the functional activities of PRMT in mammals. It also generates over 85% of the ADMA in mammals. Figure 1 illustrates the genomic structure of PRMT1.

### 2.2 Protein Structure

The protein structure of human PRMT1 shares significant homology with other members of the conserved PRMT family across eukaryotes. PRMT1 generally comprises three functional domains: the C‐terminal β‐barrel domain responsible for substrate recognition and catalysis; the N‐terminal Rossmann fold, also referred to as the S‐adenosyl‐l‐methionine (SAM) binding domain; and the α‐helical dimerization arm, essential for the establishment of an active dimeric structure. The proper performance of PRMT1's enzymatic activity within the cellular environment depends on this complex domain structure [3]. PRMT1 is associated with two recognized ligands: S‐adenosyl‐l‐homocysteine (SAH) and SAM. SAH is the byproduct of the methylation reaction, whereas AdoMet functions as the methyl donor that, catalyzed by PRMT1, transfers a methyl group to the arginine residues of the substrate protein. The methylation process is essential for numerous biological processes, including gene expression regulation and signal transduction [22].

### 2.3 | PRMT1 Isoforms

**FIGURE 2:**
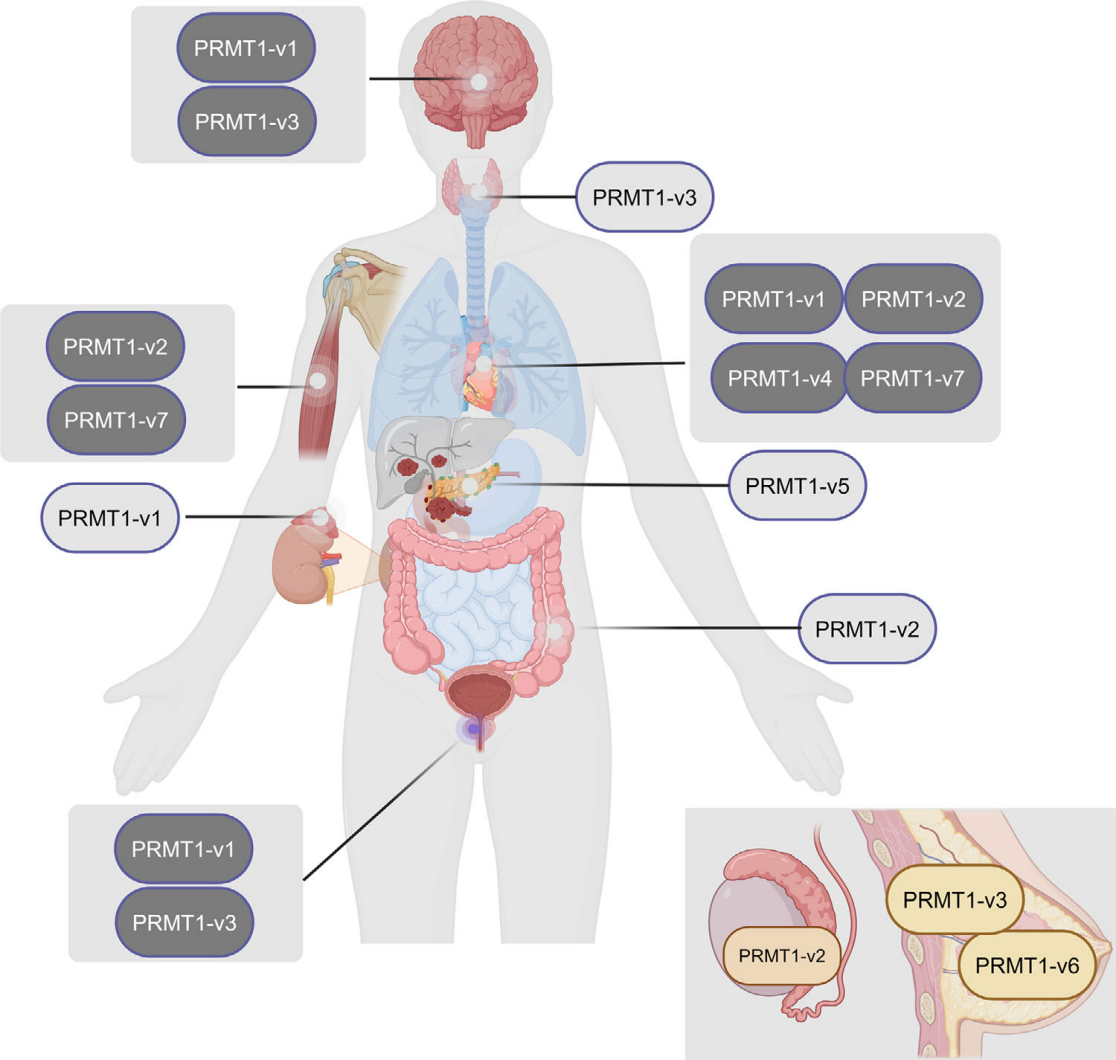
The expression of PRMT1 isoforms in organs. (1) PRMT1‐v1 demonstrates presence in the prostate, brain, heart, and adrenal glands. (2) PRMT1‐v2 demonstrates presence in the heart, skeletal muscle, colon, and testes. (3) PRMT1‐v3 demonstrates presence in the brain, cerebellum, thyroid gland, prostate, and breast. (4) PRMT1‐v4 demonstrates presence in the heart. (5) PRMT1‐v5 demonstrates presence in the pancreas. (6) PRMT1‐v6 demonstrates presence in breast cancer. (7) PRMT1‐v7 demonstrates presence in heart and skeletal muscle tissues.

Alternative splicing of the PRMT1 pre‐mRNA generates seven distinct protein isoforms, designated PRMT1‐v1 to v7. Each isoform exhibits distinct enzymatic activity and substrate selectivity, which is attributable to variations in their terminal sequences [23]. These isoforms exhibit unique, tissue‐specific expression profiles across human organs. Isoforms v1, v2, and v3 are widely expressed. PRMT1‐v1 is detected in the prostate, brain, heart, and adrenal glands, implicating it in diverse physiological processes. PRMT1‐v2 is predominantly expressed in the heart, skeletal muscle, colon, and testis, suggesting a possible involvement in muscular function and reproductive biology. PRMT1‐v3 is detected in the brain, cerebellum, thyroid, prostate, and mammary glands, suggesting its involvement in neurodevelopment and metabolic regulation. In contrast, isoforms v4 through v7 display more restricted and highly tissue‐selective expression patterns. This selective expression pattern indicates that certain isoforms may execute specialized activities in distinct tissues or participate in tissue‐specific regulation processes [21]. PRMT1‐v4 and PRMT1‐v5 exhibit selective expression, with PRMT1‐v4 localized solely in the heart and PRMT1‐v5 confined to the pancreas. This limited expression indicates that these isoforms may have specific functions in these particular organs. Moreover, PRMT1‐v7 has been detected in both cardiac and skeletal muscle tissues, suggesting a specialized role in striated muscle physiology. The specific presence of PRMT1‐v7 in striated muscle suggests a role in maintaining muscle integrity and regulating muscle‐specific functions. In contrast to the other isoforms, PRMT1‐v6 has not been detected in normal human tissues. Its expression has been observed in specific cell lines generated from breast cancer (BC), indicating a potential correlation with oncogenic mechanisms or tumor advancement. The identification of PRMT1‐v6 in BC cells suggests its involvement in the deregulation of cellular processes that drive cancer progression and indicates its potential as a therapeutic target in BC  [3]. The terminal amino acid sequence's alterations have a bearing on the enzyme's activity and its substrate recognition. Compared with PRMT1v1‐v6, PRMT1‐v7 seems to lack involvement in substrate activity regulation and is not catalytically active, while it retains the capacity to heterodimerize with other isoforms [7]. PRMT1v1‐v6 had considerable methyltransferase activity in vitro across multiple substrates, but PRMT1‐v3 and PRMT1‐v4 showed markedly reduced methylation efficacy (Figure 2).

## 3 Physiological Functions

Arginine methylation exerts an indispensable role in several biological processes, spanning the regulation of gene expression, signal transmission, RNA splicing, DNA damage response (DDR), and cell fate determination. PRMTs modulate chromatin structure and gene expression by the methylation of histone and nonhistone substrates, hence affecting the physiological and pathological states of cells.

### 3.1 RNA Splicing

RNA splicing is an essential mechanism in gene expression, during which introns are removed from pre‐mRNA and exons are ligated to form mature mRNA for translation [24]. Dysregulation of RNA splicing is a well‐established contributor to diverse pathologies, including cancer and neurological disorders [25, 26]. The catalytic substrates of PRMT1 predominantly comprise RNA‐binding proteins (RBPs) engaged in mRNA splicing and translation, distinguished by regions markedly enriched in arginine methylation. PRMT1 can modify the interactions of RBPs with precursor mRNA by methylation, hence affecting the specificity and efficiency of RNA splicing [27]. Consequently, both the depletion and overexpression of PRMT1 can induce aberrant splicing patterns, leading to disrupted gene expression and compromised cellular function. Thus, PRMT1 is implicated in the pathogenesis of cancer and neurological diseases through its role in regulating RNA splicing.

PRMT1 regulates splice site selection by methylating key splicing factors, thereby modulating their activity and RNA‐binding affinity. For instance, PRMT1‐mediated arginine methylation enhances the phosphorylation of SRSF1 and its RNA‐binding ability, promoting exon inclusion. Correspondingly, inhibition of PRMT1 reduces SRSF1 methylation, reverses aberrant splicing patterns, and suppresses cancer cell growth [28]. PRMT1 methylates SRSF1 at specific arginine residues (Arg93, Arg97, and Arg109), with Arg97 serving as the primary site. Although this methylation does not alter the subcellular localization of SRSF1, it is critical for its splicing regulatory function [29]. PRMT1 modulates the alternative splicing of viral mRNAs, thereby influencing the expression of key viral genes such as E6, E7, E1, and E2. This splicing regulation depends on PRMT1‐mediated methylation of host splicing factors, which indirectly alters the splice site selection of viral RNAs [30].

### 3.2 DNA Damage Repair

DNA damage denotes the injury and modification of DNA sequences resulting from internal and external influences within cells [31]. PRMT1 is a critical regulator of the DDR, coordinating repair pathways through the methylation of key repair proteins [32]. PRMT1 has been demonstrated to play a pivotal regulatory role in the DDR by methylating various DNA repair proteins, orchestrating the selection of homologous recombination (HR) and nonhomologous end joining (NHEJ) pathways, improving repair efficacy, and preserving genomic stability. Its aberrant function may result in repair deficiencies, genomic instability, and tumor resistance, making it a viable target for oncological therapy. A key substrate is MRE11, a core component of the MRN (MRE11–RAD50–NBS1) complex that is essential for detecting and signaling DNA double‐strand breaks (DSBs) [33]. PRMT1 methylates MRE11, a modification that is crucial for the function of both the HR and NHEJ pathways [34]. HR is regarded as an infallible repair method due to its ability to replicate homologous sequences from a template [35]. The methylation of BC type 1 susceptibility protein (BRCA1) at R610 by PRMT1 facilitates the establishment of a stable complex with BRCA1‐associated RING domain 1 (BARD1), hence aiding in HR repair. Consequently, PRMT1 depletion causes mislocalization of BRCA1 to the cytoplasm, thereby impairing HR. Moreover, PRMT1 is capable of methylating various arginine residues of hnRNPUL1, augmenting its association with NBS1, and facilitating its localization to DNA damage sites, thus aiding in DNA end resection and the HR process [36]. NHEJ is regarded as an error‐prone repair mechanism that directly re ‐ ligates DNA ends during the cell cycle with minimum resection and operates independently of a template [37]. The p53‐binding protein 1 (53BP1) is crucial in the initial processes of DNA damage detection, signaling, and repair. Boisvert et al. discovered that the glycine ‐ arginine‐rich (GAR) motif of 53BP1 is subject to methylation by PRMT1 [38]. Specifically, asymmetric dimethylation of arginines R1400, R1401, and R1403 within the 53BP1 GAR motif enhances its DNA‐binding affinity, thereby promoting NHEJ [36]. Under oxidative stress, PRMT1 can methylate APE1 at the R301 position, facilitate its mitochondrial translocation, augment cellular resistance to oxidative damage, and preserve genomic stability [39].

### 3.3 Cell Signaling

Cell signaling is a fundamental process that coordinates essential cellular activities through the transmission and interpretation of molecular cues, both extracellular and intracellular. PRMT1 influences the accuracy and efficacy of intracellular signaling by altering essential signal transduction proteins and modulating their activity and function [40]. PRMT1 is essential for various cell signaling pathways, and its aberrant expression interferes with pathways such as Akt, NF‐κB, and Wnt, leading to dysfunctional cellular activities [41, 42, 43].

The mechanistic role of PRMT1 in cellular signal transduction is primarily mediated through its catalysis of arginine methylation on specific protein substrates, thereby modulating the activity of multiple signaling pathways. PRMT1 catalyzes the methylation of the arginine residue at position 260 of estrogen receptorα (ERα), resulting in the formation of met260ERα. This methylation promotes the assembly of ERα into multiprotein complexes with PI3K, Src, and FAK, thereby activating downstream kinase cascades that drive cell proliferation and survival [44]. During erythroid differentiation, PRMT1 methylates p38α kinase at residues R49 and R149, which enhances its phosphorylation by MKK3 and subsequently activates the MAPK pathway to promote differentiation [45]. PRMT1 also directly interacts with the p65 subunit of NF‐κB, exerting a dual regulatory effect on its transcriptional activity. It can enhance the transcription of NF‐κB target genes; conversely, its methyltransferase activity can inhibit the DNA‐binding capacity of p65, thereby negatively regulating TNFα‐induced NF‐κB signaling [46]. Furthermore, PRMT1 contributes to inflammatory responses by regulating the IL‐6/STAT3 signaling axis [47]. PRMT1 also methylates cGAS at R133, thereby inhibiting its dimerization and subsequent activation of the cGAS–STING signaling pathway. This suppression dampens both type I and type II interferon responses, ultimately compromising antitumor immunity [48]. Finally, the PRMT1‐catalyzed H4R3me2a mark recruits the chromatin remodeler SMARCA4, which activates the EGFR signaling pathway to promote the proliferation and migration of colorectal cancer cells [13].

### 3.4 Cell Proliferation and Differentiation

PRMT1 has been recognized as a crucial regulator in the adult murine gut, where it orchestrates cell proliferation and differentiation [49]. Its influence encompasses the alteration of crucial transcription factors and chromatin proteins, hence orchestrating gene expression and shaping chromatin architecture, which profoundly impacts the dynamics of cell differentiation [50]. PRMT1 governs the differentiation of myoblasts into myocytes by the arginine methylation modification of the MyoD protein. MyoD is a crucial transcription factor in muscle differentiation, and PRMT1‐mediated methylation enhances myogenin gene expression, facilitating the differentiation of C2C12 cells into myocytes [51]. PRMT1 also plays a role in hematopoiesis, particularly in megakaryocyte development. Notably, inhibiting PRMT1 enhances megakaryocyte maturation, revealing a potential therapeutic strategy for myelodysplastic syndromes [52]. Similarly, PRMT1 promotes erythroid cell maturation via the p38α MAPK pathway [53]. In the immune system, PRMT1 attenuates autoimmune responses by suppressing RORγt‐dependent pathways, thereby reducing the generation of pathogenic Th17 cells [54]. The BTG2–PRMT1 complex is a crucial regulator of pre‐B cell differentiation and a suppressor of pre‐B cell leukemia [55]. Consequently, PRMT1 dysregulation is linked to impaired cellular differentiation and pathologies such as CRC [56]. Beyond differentiation, PRMT1 activity is intricately linked to the proliferative capacity of numerous cancer cells. In CRC, PRMT1 engages SMARCA4 through H4R3me2a, thereby transcriptionally activating the EGFR signaling pathway, which promotes the proliferation and migration of CRC cells [13]. In gastric cancer (GC), increased expression levels of PRMT1 correlate with augmented proliferative and metastatic capabilities of GC cells [57]. Moreover, PRMT1 inhibitors can remodel the tumor microenvironment by augmenting the quantity and functionality of CD8^+^ T cells, suppressing tumor proliferation, and restoring the efficacy of PD‐1 checkpoint blockade in resistant melanoma [58].

### 3.5 Epigenetic Regulation

PRMT1 primarily catalyzes the asymmetric dimethylation of H4R3me2a. This modification alters chromatin structure and increases chromatin accessibility, thereby facilitating gene transcription [59]. For instance, during corneal epithelial wound healing, PRMT1‐mediated H4R3me2a activates the expression of ANXA3, thereby promoting cell proliferation, migration, and accelerating wound closure [60]. Not influenced by its methyltransferase activity, PRMT1 can directly bind to SMARCC1, a core subunit of the SWI/SNF chromatin remodeling complex. This protein–protein interaction facilitates the recruitment of the SWI/SNF complex to target gene promoters, leading to transcriptional activation. In head and neck squamous cell carcinoma, this mechanism enhances IGF2BP2 transcription, thereby promoting tumor cell proliferation and chemoresistance [61]. Beyond direct chromatin regulation, PRMT1 also modulates signaling pathways by methylating nonhistone substrates, including transcription factors and signaling molecules. For example, PRMT1 catalyzes the ADMA of multiple arginine residues on β‐catenin. This modification impairs the interaction between β‐catenin and the destruction complex, thereby inhibiting its ubiquitination and degradation. Consequently, methylated β‐catenin accumulates in the cytoplasm and translocates to the nucleus, where it associates with TCF/LEF coactivators to upregulate target genes such as c‐Myc and cyclin D1, thereby driving cell proliferation and tumorigenesis [57, 62].

## 4 The Role of PRMT1 in Cancer

PRMT1 functions as a principal regulator of arginine methylation, critically driving malignant tumor progression via diverse molecular mechanisms. PRMT1 promotes proproliferative gene transcription by catalyzing the H4R3me2a mark, modulates key transcription factors including E2F1, STAT3, and HIF‐1α, and impacts critical cellular processes such as the DDR, cell cycle progression, and apoptosis. Furthermore, PRMT1 facilitates epithelial–mesenchymal transition (EMT) via the upregulation of pathways such as ZEB1 and TGF‐β/Smad, promotes tumor angiogenesis by regulating vascular endothelial growth factor (VEGF), and contributes to chemotherapy resistance by modifying metabolic enzymes and DNA repair proteins. However, PRMT1 function is context dependent can be tumor‐suppressive in specific cancer types. Despite the challenges in developing PRMT1‐targeted therapies, combination strategies employing PRMT1 inhibitors with PARP inhibitors (PARPis) or immunotherapeutic agents demonstrate considerable potential to overcome drug resistance and remodel the tumor microenvironment.

### 4.1 Molecular Mechanisms of PRMT1 in Cancer

#### 4.1.1 PRMT1 Functions in Cancer by Regulating Gene Expression

The most recognized substrate of PRMT1 is H4R3, and the H4R3me2a methylation mediated by PRMT1 serves as a significant signal for transcriptional activity [63]. The H4R3me2a mark on chromatin is identifiable by specific domains, such as the Tudor domain, within various transcriptional coactivators or chromatin remodeling complexes. This recognition facilitates the recruitment of these factors to gene promoter or enhancer regions, promoting chromatin accessibility, establishing conducive conditions for the binding of the transcriptional machinery, and ultimately initiating the expression of downstream genes [64]. PRMT1 facilitates tumor growth by enriching the H4R3me2a mark at the promoters of proproliferative genes to enhance their transcription. Transcription factor E2F1 is a crucial component in the regulation of the cell cycle. The PRMT1‐mediated dimethylation of E2F1 at R109 enhances its DNA‐binding affinity, upregulates S‐phase kinases like cyclin A2 and CDK2, and promotes S‐phase entry [65]. Beyond transcription factors, PRMT1 also regulates the m^6^A RNA methyltransferase METTL14. It catalyzes the asymmetric dimethylation of METTL14 at arginines 442 and 445 within its C‐terminal RGG motif, thereby enhancing METTL14's activity and promoting tumor cell proliferation and transformation. This methylation alteration is recognized and bound by SPF30, while Jmjd6 can reverse it. The PRMT1 inhibitor MS023 effectively inhibits METTL14 methylation and markedly reduces its capacity to promote cancer cell proliferation and clonogenicity, suggesting that PRMT1 is pivotal in carcinogenesis through the regulation of METTL14's PTM [66].

Additionally, PRMT1 methylates and activates key transcription factors in critical signaling pathways, such as NF‐κB [46], STAT3 [67], and HIF‐1α [68], therefore influencing cancer progression. For instance, PRMT1 methylates STAT3 by catalyzing its ADMA at arginine 688, which enhances STAT3's transcriptional activity and expression. Consequently, the binding of TIPE1 to the PRMT1 catalytic domain inhibits this methylation event, attenuating STAT3 activity and thereby impeding the malignant progression of osteosarcoma [67]. In Fms‐like receptor tyrosine kinase 3 (FLT3)‐ITD mutant acute myeloid leukemia (AML), PRMT1 expression is markedly elevated. PRMT1 specifically binds to the FLT3‐ITD mutant protein and catalyzes its asymmetric dimethylation at arginine residues 972 and 973. This modification enhances phosphorylation at FLT3 tyrosine 969, promotes the recruitment of SH2 domain‐containing proteins such as GRB2, and leads to sustained activation of downstream survival pathways, including STAT5 and AKT, thereby sustaining leukemic cell survival and proliferation [69]. Li et al. [68] reported that both PRMT1 expression and its‐mediated methylation of hypoxia‐inducible factor 2β (HIF2β) were markedly elevated in BC. Under hypoxic conditions, PRMT1 was identified as a direct transcriptional target of HIF1α. Furthermore, PRMT1 augmented the expression of HIF1α‐driven glycolytic genes. Mechanistically, PRMT1‐mediated methylation of HIF2β at arginine 42 facilitated the formation of HIF1α/HIF2β heterodimers, enhanced their chromatin binding, and potentiated their transcriptional activity [68]. In hepatocellular carcinoma (HCC), PRMT1 directly enhances SOX18 transcription by increasing H4R3me2a levels at its promoter, thereby promoting HCC cell proliferation [70]. Similarly, in laryngeal cancer, PRMT1 modulates nuclear receptor coactivator 5 (NCOA5) expression via H4R3me2a, facilitating tumor cell proliferation, migration, and invasion [71]. In CRC, PRMT1‐mediated methylation of phosphoglycerate kinase 1 (PGK1) augments its ERK‐mediated phosphorylation, which activates downstream signaling to drive glycolysis and carcinogenesis [72]. Wang et al. [57] demonstrated that KTN1 inhibited K48‐linked ubiquitination facilitated by the E3 ubiquitin ligase TRIM48 in the cytoplasm by binding to PRMT1, thereby obstructing the proteasomal degradation of PRMT1 and preserving its protein stability. In the nucleus, stable PRMT1 recruited the transcription factor MLXIP, which, in conjunction, bound to the β‐catenin promoter region to form a transcriptional activation complex, significantly enhancing the expression of β‐catenin and its downstream target genes (such as MYC, CCND1, TCF1, etc.), thereby perpetuating the activation of the β‐catenin signaling pathway and ultimately fostering the proliferation, migration, invasion, and in vivo metastasis of GC cells [57]. In the BC microenvironment, IL‐6 released by tumor‐associated macrophages initiates PRMT1‐mediated asymmetric dimethylation of enhancer of zeste homolog 2 (EZH2) at arginine 342 [73]. This methylation event promotes PRC2 assembly by enhancing the EZH2–SUZ12 interaction. Consequently, transcription of P16 and P21 is suppressed due to enhanced EZH2 activity and increased H3K27me3 deposition at their promoters. Thus, elevated EZH2 levels facilitate BC cell metastasis by repressing metastasis‐suppressor genes such as E‐cadherin, DAB2IP, and CSTA. Separately, the reduction in P16 and P21 levels promotes cell cycle progression and proliferation [74].

#### 4.1.2 PRMT1 Plays a Role in Cancer by Regulating the Cancer Cell Cycle and Apoptosis

The dysregulation of the cell cycle and the suppression of apoptosis are fundamental characteristics of cancer, and PRMT1 is a significant contributor to both processes. PRMT1 promotes proliferation primarily by facilitating the G1 to S phase transition. For instance, PRMT1‐catalyzed asymmetric dimethylation of E2F1 at R109 (E2F1–R109me2a) enhances its affinity for target gene promoters, upregulates S‐phase kinases like cyclin A2 and CDK2, and facilitates S‐phase entry. Concurrently, PRMT1 upregulates cyclin D1 expression through H4R3me2a modification. Furthermore, as a component of the C/EBPα complex, PRMT1 methylates C/EBPα at three arginine residues (R35, R156, and R165). This methylation enhances cyclin D1 production by disrupting the interaction between C/EBPα and its corepressor HDAC3, thereby accelerating tumor cell proliferation in BC [75]. Beyond cell cycle control, PRMT1 also drives the progression and drug resistance of multiple myeloma by modulating key pathways involved in cell cycle regulation, apoptosis, and immune evasion. Its elevated expression is closely associated with relapsed/refractory illnesses, highlighting its potential as a therapeutic target. Accordingly, pharmacological inhibition of PRMT1 with compounds such as MS023 directly eliminates multiple myeloma cells and synergizes with immunotherapy, presenting a novel therapeutic strategy for refractory disease [76].

A primary mechanism by which PRMT1 inhibits apoptosis is through the direct regulation of the pivotal tumor suppressor p53 [77]. p53 is a master regulator of apoptosis, genomic integrity, and tumor suppression. Consequently, the functional loss of p53 is a major driver of tumorigenesis and therapy resistance, making the restoration of its activity a central goal in oncology. For instance, in BC, PRMT1 directly methylates p53, impairing its transcriptional activity and preventing the transactivation of downstream proapoptotic genes, thereby facilitating tumor progression [78]. Similarly, in retinoblastoma, elevated PRMT1 expression promotes Y79 cell proliferation via the p53/p21/CDC2/cyclin B pathway, whereas PRMT1 silencing inhibits cell growth [79]. In non‐small cell lung cancer (NSCLC), PRMT1 promotes the Warburg effect, and its stability is regulated by USP7. Interestingly, p53 itself acts as a glucose sensor that suppresses glycolysis and proliferation by regulating the USP7–PRMT1 axis, suggesting this pathway as a therapeutic target for NSCLC patients regardless of p53 status [12]. While elevated PRMT1 typically correlates with a poor prognosis in many cancers, the opposite is true in non‐MYCN‐amplified neuroblastoma, where its loss predicts worse outcomes. Here, PRMT1 exerts a tumor‐suppressive function by activating the p53/p21/PAI‐1 pathway, thereby promoting cellular senescence and inhibiting migration. This demonstrates the context ‐ dependent, dual nature of PRMT1 in cancer and indicates that universal therapeutic targeting may be ineffective [80].

PRMT1 is also a critical regulator of the DDR. The MRE11–RAD50–NBS1 complex is crucial for cellular damage repair. The complex can be found at the locus of DNA damage during the first phase. Specifically, PRMT1 methylates MRE11 at arginine residues within its C‐terminal GAR motif, a modification essential for proper DNA damage checkpoint activation [34, 81]. Beyond direct protein methylation, PRMT1 maintains genomic integrity in clear cell renal cell carcinoma by modulating RNA metabolism, including the splicing and expression of DDR‐related genes. Consequently, PRMT1 inhibition disrupts DNA repair, leading to R‐loop accumulation, increased DNA damage, and ultimately, cell death [82]. A similar reliance on PRMT1‐mediated regulation of RNA metabolism and the DDR has been shown to sustain pancreatic ductal adenocarcinoma [83]. Wang et al. [84] investigated the correlation between cellular energy consumption and DNA repair mechanisms. Under energy‐deficient conditions, ATG4B translocates to the nucleus, binds to PRMT1, and inhibits its methylation of MRE11. This impairs the DDR and promotes genomic instability. Conversely, inhibiting ATG4B restores PRMT1‐mediated MRE11 methylation, enhances the DDR, reduces mutation load, and significantly prolongs survival in a murine AML model [84].

#### 4.1.3 PRMT1 Plays a Role in Cancer by Regulating EMT

EMT is a critical process that confers invasive and metastatic capabilities upon tumor cells through morphological alterations, loss of cell–cell adhesion, and enhanced motility [85]. PRMT1 is a well‐established promoter of EMT across various malignancies. For instance, in renal tubular epithelial cells under high glucose conditions, elevated PRMT1 expression correlates with a canonical EMT marker shift: downregulation of E‐cadherin and upregulation of N‐cadherin, Vimentin, and α‐SMA. This indicates its role in promoting the EMT process, with PRMT1 potentially activating the classical profibrotic pathway through the enhancement of TGF‐β secretion and phosphorylation of SMAD3. TGF‐β/SMAD3 further enhances the expression of downstream EMT transcription factors (SNAIL/SLUG), establishing a positive feedback loop [86]. Xiong et al. [87] identified that PRMT1 can facilitate the arginine methylation and phosphorylation of BRD4, hence promoting EMT. Similarly, in liver cancer, PRMT1 knockdown reverses the EMT phenotype, downregulating mesenchymal markers (Vimentin, Snail, N‐cadherin) and upregulating E‐cadherin. Furthermore, PRMT1 knockdown downregulates TGF‐β1 and phospho‐Smad2/3, while its overexpression has the opposite effect, confirming that PRMT1 facilitates EMT in liver cancer via the TGF‐β1/Smad pathway and highlighting its therapeutic potential [88]. Zhang et al. [62] demonstrated that PRMT1 downregulates the epithelial marker E‐cadherin and upregulates the mesenchymal markers N‐cadherin, Vimentin, Snail, and βcatenin in GC, thereby promoting the EMT. Subsequent investigations revealed that PRMT1 silencing promotes LATS1 phosphorylation, which in turn induces YAP phosphorylation. Conversely, PRMT1 overexpression suppressed the phosphorylation of both LATS1 and YAP. These findings indicate that PRMT1 facilitates EMT by inhibiting the Hippo signaling pathway, highlighting its critical role in GC progression [62]. In NSCLC, PRMT1 regulates EMT by methylating the transcription factor TWIST1, thereby promoting disease progression [89]. In BC models, PRMT1 facilitates the asymmetric dimethylation of H4R3me2a, is recruited to the ZEB1 promoter, and directly activates ZEB1 transcription. Increased ZEB1 subsequently induces EMT, resulting in cancer cells losing polarity and adhesion, adopting a mesenchymal phenotype, and acquiring improved migratory and invasive abilities, hence facilitating metastasis. Conversely, PRMT1 inhibition or depletion leads to diminished ZEB1 expression, suppression of the EMT program, and reduced cellular invasiveness. Concurrently, it induces a marked senescent phenotype, characterized by cell cycle arrest at the G1 phase, the emergence of tetraploid DNA, upregulation of p21, downregulation of G2/M‐phase genes (e.g., cyclin A2, cyclin B1, CDK1), and positive senescence‐associated β‐galactosidase staining, ultimately inhibiting proliferative capacity. Thus, PRMT1 governs BC progression through a dual mechanism. It either activates ZEB1 to facilitate EMT and metastasis or, upon its inhibition, triggers senescence to suppress proliferation. This mechanistic duality provides a rationale for therapeutic strategies targeting the PRMT1–ZEB1 axis [90].

#### 4.1.4 PRMT1 Exerts its Oncogenic Function by Regulating Angiogenesis

Diverse angiogenic agents collaboratively promote endothelial cell proliferation, resulting in enhanced vascularity. Angiogenesis facilitates tumor growth and progression, including BC, by stimulating the production of new blood vessels to satisfy the metabolic demands of multiplying cancer cells [91, 92]. When a solid tumor reaches a critical volume (typically 1–2 mm^3^), it becomes dependent on inducing angiogenesis to maintain its oxygen and nutrient supply [93]. PRMT1 is a key facilitator of tumor angiogenesis, primarily through its regulation of critical proangiogenic factors. Among these, VEGF is the most pivotal [94]. In BC, PRMT1's modulation of VEGF is mostly associated with the cellular response to hypoxia. Rapidly proliferating cancer cells within the tumor mass frequently create local hypoxic conditions, leading to the stabilization and activation of HIF‐1α. HIF‐1α is a master transcriptional regulator of numerous proangiogenic genes, including VEGF. PRMT1 functions as a crucial coactivator within the HIF‐1α signaling pathway. Under hypoxia, PRMT1 is recruited to the promoters of HIF‐1α target genes, where its deposition of the H4R3me2a mark enhances transcriptional activity, leading to the robust upregulation of VEGF expression and secretion [68].

### 4.2 The Role of PRMT1 in Cancer Therapy

Beyond its roles in tumor initiation and progression, PRMT1 is increasingly recognized as a central driver of therapeutic resistance across multiple cancer types, though this understanding has yet to be fully translated into clinical applications. For instance, CRC, PRMT1‐mediated methylation of metabolic enzymes like PGK1 and phosphoglycerate dehydrogenase (PHGDH) enhances glycolysis and serine synthesis, promotes the Warburg effect, and increases tumor stemness, thereby fostering a stress‐adaptive state conducive to resistance [72]. Similarly, PRMT1 can suppress the cGAS–STING‐I type interferon pathway in GC, impede M1 macrophage polarization and CD8^+^ T cell recruitment triggered by dsDNA, and preserve the “cold tumor” phenotype [95]. In triple‐negative BC, PRMT1 promotes resistance by methylating targets such as EGFR, PARP1, and PHGDH, thereby augmenting DNA damage repair and prosurvival signaling. Consequently, PRMT1 expression correlates with metastatic potential, and its inhibition can restore docetaxel sensitivity and suppress distant metastasis [96]. In CRC, elevated PRMT1 expression is positively associated with meR206–PGK1 and pS203–PGK1, indicating a bad prognosis, and it may serve as a combination biomarker to inform the application of methyltransferase inhibitors [72]. Furthermore, PRMT1 confers ferroptosis resistance in CRC by catalyzing H4R3me2a to activate transcription of solute carrier family 7 member 11 (SLC7A11). This process is negatively regulated by LPCAT2, which acetylates PRMT1 at K145 to sequester it in the cytoplasm, thereby inhibiting SLC7A11 activation and restoring ferroptosis sensitivity [97]. PRMT1 also methylates RIP3 at arginine 486, thereby inhibiting the interaction between RIP3 and RIP1, which suppresses necrosome formation and downstream necroptotic signaling, eventually limiting tumor immune evasion [98]. Additional mechanisms include PRMT1‐mediated methylation of PKP2 and SOX2, which contributes to radioresistance and chemoresistance in lung cancer [99, 100]. In FLT3‐ITD mutant AML, genetic or pharmacological suppression of PRMT1 markedly decreases FLT3‐ITD methylation levels and improves the effectiveness of tyrosine kinase inhibitors in eradicating leukemia progenitor cells. This indicates that PRMT1‐mediated FLT3 methylation is an essential mechanism for the preservation and treatment resistance of FLT3‐ITD AML. The dual suppression of PRMT1 and FLT3 is anticipated to surmount TKI resistance and enhance patient outcomes [69].

While DDR helps preserve genomic integrity in normal cells, it may provide resistance to DNA damage caused by radiotherapy and chemotherapy in cancer cells, leading to drug resistance and highlighting the possible involvement of PRMT1 in therapeutic resistance in tumors [101]. In BC, PRMT1 can be triggered by radiotherapy, leading to the methylation of BRCA1, which facilitates its association with BARD1 and its location in the nucleus, so effectively beginning HR repair, blocking apoptosis, and increasing resistance to PARPis such as olaparib [102]. In NSCLC, PRMT1 methylates desmoplakin PKP2, binds the deubiquitinase USP7, and stabilizes β‐catenin, which upregulates the expression of the NHEJ crucial factor LIG4, consequently increasing DNA DSB repair and resulting in radiation resistance [103]. In pancreatic cancer, PRMT1 expression increases after gemcitabine (GEM) treatment, with acquired chemoresistance promoted by delayed clearance of DNA damage markers (e.g., γ H2AX and RPA32), prolonged S‐phase arrest, and improved damage repair mechanisms. Consequently, PRMT1 inhibition significantly sensitizes pancreatic cancer cells to GEM [104]. In summary, PRMT1 functions as a master epigenetic regulator of multiple DNA repair pathways, enabling tumor cells to survive genotoxic stress from cancer therapies. Targeting PRMT1, therefore, represents a promising strategy to overcome this key mechanism of therapeutic resistance.

Consequently, PRMT1 emerges not only as a promising prognostic biomarker but also as a compelling therapeutic target. Combining PRMT1 inhibition with established modalities presents a novel strategic avenue to overcome drug resistance and suppress metastasis, thereby addressing critical challenges in oncology.

### 4.3 Challenges and Future Directions

As with other targeted therapies, the development of resistance to PRMT1 inhibitors through various mechanisms is a foreseeable challenge. Proactively developing combination therapies is therefore critical to overcoming resistance and improving therapeutic efficacy. The involvement of PRMT1 in DDR indicates that the concurrent application of the PRMT1 inhibitor MS023 with a PARPi or conventional chemotherapeutic agents can markedly elevate DNA damage levels and diminish cell viability [105]. Similarly, low‐dose GSK3368715, a specific PRMT1 inhibitor, acts synergistically with olaparib or rucaparib to overcome PARPi resistance in both in vitro and in vivo models [106]. This approach is also effective in MTAP‐deficient NSCLC, where PRMT1 inhibition mitigates PARPi resistance and significantly enhances its cytotoxicity [107]. Furthermore, the inhibition of PRMT1 can elevate PD‐L1 expression, activate the type I interferon pathway, and augment macrophage and T cell infiltration. The combination of PRMT1 inhibitors with anti‐PD‐1/PD‐L1 immunotherapy can synergistically modify the tumor immune microenvironment and bolster the antitumor immune response [108]. In GC, the knockdown of PRMT1 suppresses the cGAS/STING pathway, leading to the generation of type I interferon and facilitating M1‐like macrophage polarization within the tumor microenvironment [95]. Considering that PRMT1 may modulate the expression of immune molecules, the integration of its inhibitors with immunotherapy demonstrates significant potential.

**FIGURE 3:**
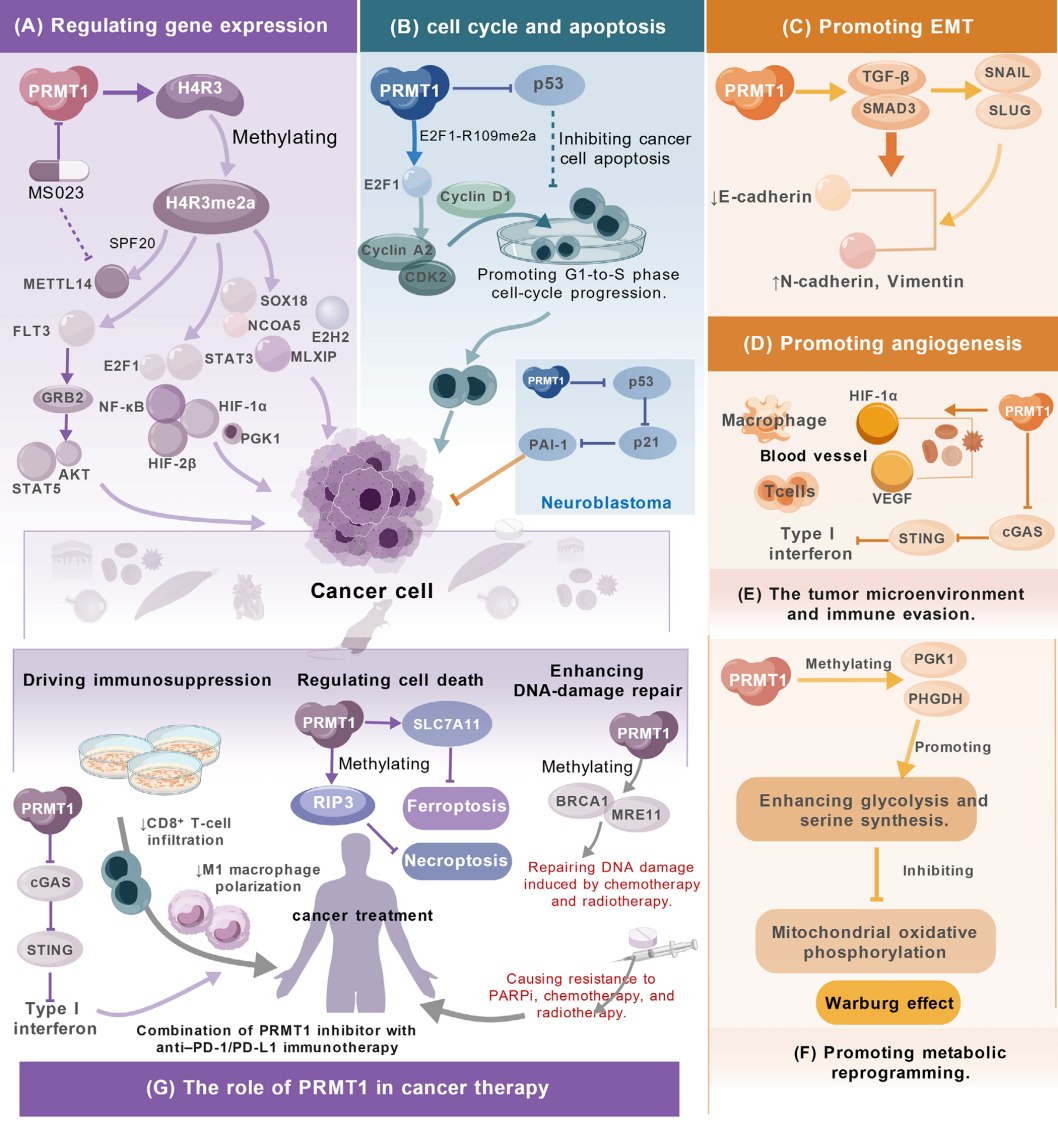
The role of PRMT1 in cancer. (A) The role of PRMT1 in regulating gene expression. (B) The role of PRMT1in cell cycle and apoptosis. (C) The role of PRMT1 in epithelial–mesenchymal transition. (D) The role of PRMT1 in angiogenesis. (E) The role of PRMT1 in tumor microenvironment and immune evasion. (F) The role of PRMT1 in promoting metabolic reprogramming. (G) The role of PRMT1 in cancer therapy.

In summary, PRMT1 is a master regulator of oncogenesis, driving tumor initiation and progression through the precise epigenetic control of fundamental cellular processes such as gene transcription, cell cycle progression, apoptosis, EMT, and angiogenesis. Despite the therapeutic challenges in targeting it, the ongoing development of selective inhibitors and an enhanced comprehension of its actions provide PRMT1‐directed methods, particularly combination therapies, a potential new approach for addressing a wide range of malignant tumors. The role of PRMT1 in cancer is presented in Table 1 and illustrated in Figure 3.

**TABLE 1:** The role of PRMT1 in cancer.

Cancer	Mechanism	Molecules/pathways	Influence	References
Breast cancer	Methylating E2F1 at R109 enhances its promoter‐binding affinity and accelerates S‐phase entry; methylating C/EBPα upregulates cyclin D1 transcription; methylating of p53 suppresses its trans‐activation capacity; HIF‐1α‐mediated induction of VEGF; methylating of EZH2 at R342 silences p16/p21 expression and thereby promotes metastasis.	E2F1, C/EBPα, p53, HIF‐1α, VEGF, EZH2, ZEB1	Promoting proliferation, metastasis, angiogenesis, and immune evasion	[68, 74, 75, 90]
Acute myeloid leukemia	Methylating FLT3‐ITD at R972/R973 enhances its phosphorylation and activates the STAT5/AKT pathway.	FLT3‐ITD, STAT5, AKT	Promoting leukemia cell survival, proliferation, and drug resistance	[69]
Hepatocellular carcinoma	Upregulating SOX18 expression	SOX18	Promoting proliferation	[70]
Laryngeal carcinoma	Regulating NCOA5 expression	NCOA5	Promoting proliferation, migration, and invasion	[71]
Colorectal cancer	Methylating PGK1 enhances ERK phosphorylation and promotes glycolysis; activating SLC7A11 and thereby suppresses ferroptosis.	PGK1, SLC7A11	Promoting glycolysis, proliferation, and drug resistance	[72, 97]
Gastric cancer	KTN1 binds PRMT1 and prevents its degradation, thereby potentiating β‐catenin signaling; suppressing the cGAS–STING pathway to sustain a “cold‐tumor” phenotype.	β‐Catenin, cGAS–STING and LATS1/YAP (Hippo pathway)	Promoting proliferation, metastasis, and immune evasion	[109]
Non‐small cell lung cancer	Methylating TWIST promotes EMT; methylating PKP2 stabilizes β‐catenin, upregulates LIG4, and enhances DNA repair.	TWIST, PKP2, β‐catenin, LIG4	Promoting EMT and radioresistance	[89, 99, 100, 103]
Osteosarcoma	TIPE1 suppresses PRMT1‐mediated methylation of STAT3, thereby diminishing its activity.	STAT3	Inhibiting malignant progression	[67]
Multiple myeloma	Regulating cell‐cycle, apoptotic, and immune–evasion pathways	—	Promoting tumor progression and drug resistance	[76]
Retinoblastoma	Promoting proliferation via the p53/p21/CDC2/cyclin B pathway	p53 pathway	Promoting proliferation	[79]
Neuroblastoma	In non‐MYCN‐amplified tumors, it suppresses the p53/p21/PAI‐1 axis, thereby inhibiting senescence and migration.	p53 pathway	Inhibiting tumor progression	[80]
Pancreatic cancer	Promoting DDR, delaying γH2AX/RPA32 clearance, and potentiating gemcitabine resistance	DDR	Promoting chemoresistance	[104]
Renal cell carcinoma	Regulating RNA metabolism and splicing of DDR‐related genes	RNA metabolism, DDR	Maintaining genomic integrity	[82]
Triple‐negative breast cancer	Methylating EGFR, PARP1, and PHGDH to enhance DNA damage repair and prosurvival signaling	EGFR, PARP1, PHGDH	Promoting lung metastasis and docetaxel resistance	[96]

## 5 The Role of PRMT1 in Other Diseases

### 5.1 The Role of PRMT1 in Chronic Liver Pathologies

**FIGURE 4:**
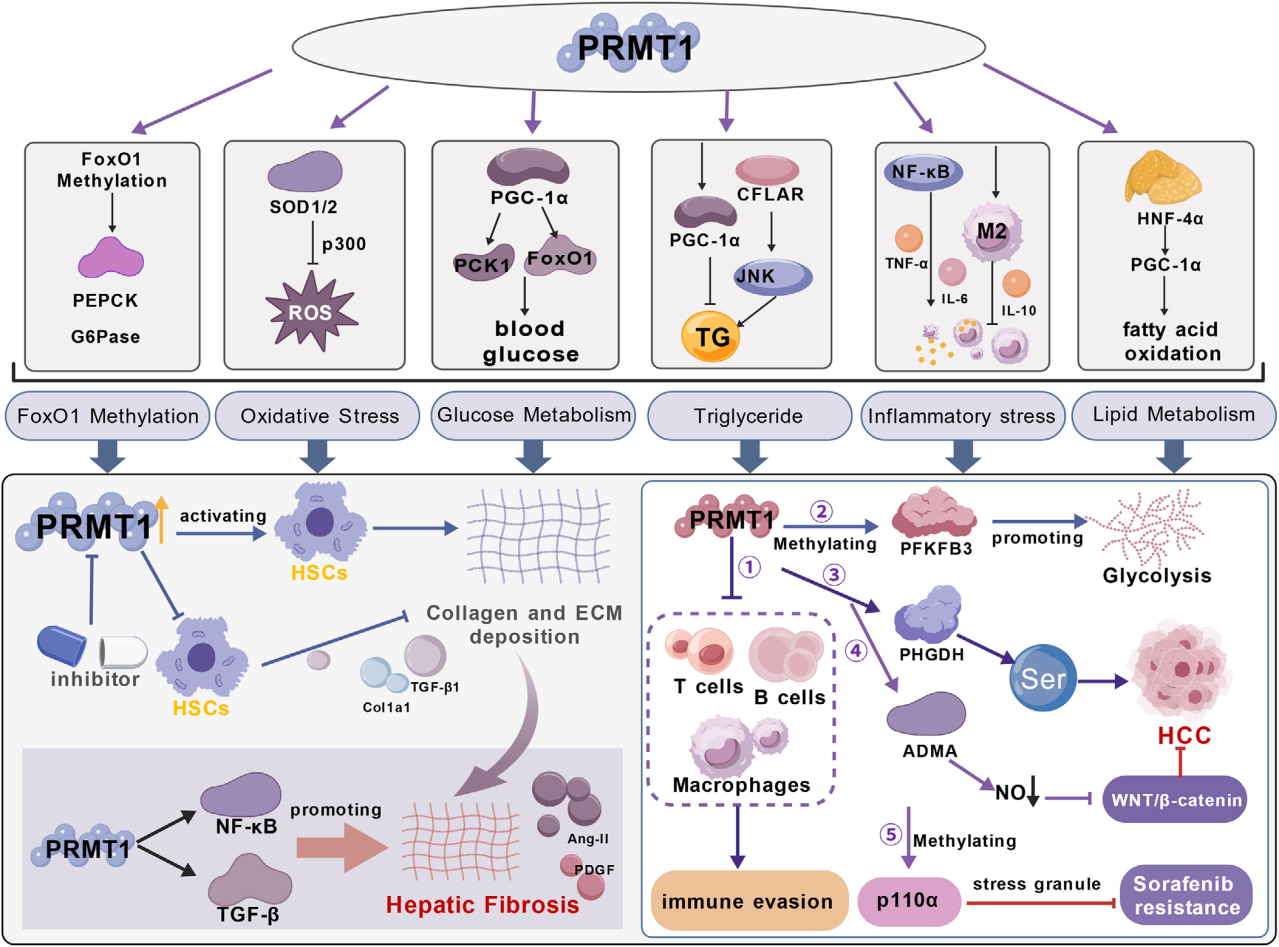
The role of PRMT1 in chronic liver pathologies. (A) PRMT1 can regulate the occurrence and development of NAFLD through various pathways, including the regulation of FoxO1 methylation modification, oxidative stress, glucose metabolism, triglyceride homeostasis, modulation of inflammatory responses, and lipid metabolism. (B) The activation of HSCs and the excessive deposition of ECM are the main characteristics of liver fibrosis. PRMT1 can activate hepatic stellate cells and inflammatory signaling pathways, thereby exacerbating the progression of liver fibrosis. (C) The role of PRMT1 in HCC: (1) PRMT1 reduces immune cells in the tumor microenvironment. (2) PRMT1 methylates PHGDH to promote serine synthesis, thereby supporting tumor growth. (3) PRMT1 methylation enhances the activity of PHGDH, thereby promoting serine production. (4) PRMT1 leads to increased nitric oxide production by generating ADMA and inhibiting nitric oxide synthase, ultimately inducing oxidative stress and inflammatory responses. (5) PRMT1 impedes the role of p110α in promoting stress granule (SG) assembly by facilitating the arginine methylation of the phosphatidylinositol 3‐kinase catalytic subunit α (p110α), thereby mitigating the resistance of HCC to sorafenib by inhibiting the formation of SGs.

CLDs include several disorders affecting more than 300 million people worldwide, mostly due to alcohol abuse, chronic viral hepatitis, and metabolic disorders such as NAFLD. A hallmark of CLDs is a prolonged asymptomatic phase that can persist for decades without clinical manifestation. The disease progression involves complex pathological alterations at the cellular and molecular levels, driven by persistent liver injury from various detrimental stimuli.  [110]. Along with a rising frequency among teenagers, CLDs and their complications—including cirrhosis and HCC—contribute significantly to the burden of mortality, morbidity, and economic burden [111]. Currently, no curative therapies exist for CLDs, underscoring the critical need to identify viable therapeutic targets. Emerging evidence now implicates PRMT1 as a critical driver of CLD progression, suggesting that PRMT1‐mediated protein methylation is a pivotal mechanism influencing the pathogenesis of chronic hepatic disorders (Figure 4).

#### 5.1.1 The Role of PRMT1 in NAFLD

NAFLD is a chronic hepatic condition characterized by excessive lipid accumulation inside hepatocytes, arising in the absence of elevated alcohol consumption or other discernible etiologies of hepatic impairment. It is strongly associated with metabolic syndrome, including central adiposity and insulin resistance, which are key drivers of its pathogenesis [112]. The disease encompasses a histological spectrum, ranging from simple steatosis to NASH, which is characterized by lobular inflammation and hepatocyte ballooning, and can progress to fibrosis, cirrhosis, and HCC [113]. The pathophysiology of NAFLD is intricate and not completely elucidated. The prevailing perspective is that it arises from the interaction of various factors, encompassing insulin resistance, oxidative stress, endoplasmic reticulum (ER) stress, inflammatory responses, dysbiosis of gut microbiota, mitochondrial dysfunction, iron overload, adipose tissue dysfunction, and genetic and epigenetic influences [114, 115].

##### 5.1.1.1 PRMT1 Plays a Role in NAFLD by Regulating the Methylation Modification of FoxO1

The transcription factor Forkhead box protein O1 (FoxO1) is a pivotal regulator of NAFLD progression, primarily through its control of hepatic lipid and glucose homeostasis. FoxO1 modulates the expression of key genes such as sterol regulatory element‐binding protein 1c and the *Fas* cell surface death receptor, directly influencing fatty acid synthesis and triglyceride (TG) accumulation [116, 117]. In NAFLD animal models, FoxO1 activity has been demonstrated to be essential for maintaining lipid homeostasis, underscoring its beneficial metabolic functions [118]. Pharmacological inhibition of FoxO1 with compounds like AS1842856 downregulates these target genes, reduces hepatic lipid accumulation, and consequently ameliorates steatosis [119]. Beyond metabolism, FoxO1 is implicated in critical stress and cell death pathways, including the regulation of ER stress and necroptosis, which are central to NAFLD pathogenesis [120]. FoxO1 activation exacerbates both ER stress and necroptosis, whereas its inhibition attenuates these pathological processes, highlighting its potential as a therapeutic target. FoxO1 also contributes to hepatic inflammation, a key driver of NAFLD progression, by directly regulating the transcription of proinflammatory mediators like tumor necrosis factor‐alpha and interleukin‐1β [121]. Moreover, FoxO1 is prepared to interact with additional signaling pathways, including the SIRT3/FOXO1 axis, to jointly regulate NAFLD progression. SIRT3 can modulate FoxO1 activity through deacetylation, potentially impacting the progression of NAFLD [122]. Thus, through its integral role in hepatic lipid metabolism, inflammatory signaling, and the regulation of ER stress and cell death pathways, FoxO1 and its associated regulatory network emerge as a compelling therapeutic target for NAFLD.

PRMT1 has been identified as a methylator of FoxO1, hence influencing its activity and affecting its role in the control of hepatic glycogen [123]. Insulin inhibits FoxO1 activity in the liver by increasing Akt (protein kinase B), thereby diminishing gluconeogenesis [124]. PRMT1, by the methylation of FoxO1, may impair the inhibitory influence of the insulin/Akt signaling pathway on FoxO1, leading to heightened FoxO1 activity in the liver. Consequently, this aberrant FoxO1 activation promotes the transcription of key gluconeogenic enzymes, such as phosphoenolpyruvate carboxykinase and glucose‐6‐phosphatase, exacerbating fasting hyperglycemia and insulin resistance in NAFLD [125]. Beyond gluconeogenesis, PRMT1 also impairs hepatic glycogen synthesis via FoxO1 methylation. Normally, FoxO1 promotes glycogen synthesis in response to low blood glucose. The methylation of FoxO1 by PRMT1 may modify its subcellular location and transcriptional activity, thereby influencing glycogen production and breakdown. This may potentially worsen insulin resistance and glycemic control problems in patients with NAFLD [126].

##### 5.1.1.2 PRMT1 Contributes to NAFLD Through Modulating Oxidative Stress Responses

It was indicated that PRMT1 plays a regulatory role in the expression of antioxidant genes in the context of alcoholic liver disease. Specifically, PRMT1 directly binds to and activates the promoters of antioxidant genes such as SOD1 and SOD2, facilitating the recruitment of the p300 acetyltransferase to enhance their transcription and attenuate oxidative stress [127]. A role for PRMT1 in regulating oxidative stress has also been implicated in NAFLD, although its function appears more complex. Elevated hepatic PRMT1 levels are observed in obese mice as a consequence of a high‐fat diet (HFD) regimen, while its methyltransferase activity is reduced. The discrepancy between expression and activity indicates a multifaceted role for PRMT1 in hepatic disorders. Subsequent research has revealed that downregulating PRMT1 intensifies hepatic steatosis in mice, whereas overexpressing wild‐type PRMT1 alleviates liver fat accumulation caused by a HFD. The protective effect relies on the methyltransferase activity of PRMT1, which increases fatty acid oxidation by increasing the production of peroxisome proliferator‐activated receptor gamma coactivator 1‐alpha (PGC‐1α) mRNA, a crucial transcriptional coactivator that governs mitochondrial biogenesis and energy metabolism. Mechanistically, PRMT1 enhances PGC‐1α expression by methylating the transcription factor HNF‐4α, which increases its binding affinity to the PGC‐1α promoter and boosts PGC‐1α transcription. Corroborating these findings, diminished expression of both PRMT1 and PGC‐1α in the livers of obese patients correlates with the severity of hepatic steatosis. Collectively, these data establish a protective role for PRMT1 in NAFLD, whereby its methyltransferase activity activates the HNF‐4α/PGC‐1α pathway to enhance fatty acid oxidation and mitigate steatosis. These findings identify the PRMT1/HNF‐4α/PGC‐1α axis as a promising therapeutic target [17]. In summary, while direct evidence implicating PRMT1 in the regulation of oxidative stress in NAFLD remains limited, insights from studies conducted in both ALD and NAFLD models indicate that PRMT1 may influence oxidative stress through multiple mechanisms, including the modulation of antioxidant gene expression and functional synergy with PGC‐1α. Further research is necessary to fully elucidate the specific mechanisms by which PRMT1 operates in the context of NAFLD.

##### 5.1.1.3 PRMT1 Exerts its Function in NAFLD by Controlling Glucose Metabolism

PRMT1 plays a critical role in hepatic glucose metabolism, skeletal muscle atrophy, and lipolytic metabolism in adipocytes [128]. Murine models of diabetes, established through a HFD or streptozotocin administration, exhibit significant upregulation of the hepatic PRMT1‐v2 isoform. This upregulation correlates with enhanced gluconeogenic capacity and hyperglycemia. Conversely, hepatocyte‐specific deletion of PRMT1 attenuates gluconeogenesis and ameliorates hyperglycemia, confirming its pathogenic role. Ma et al. [129] further demonstrated that the nuclear‐enriched PRMT1‐v2 splice variant directly interacts with the transcriptional coactivator PGC‐1α and enhances its activity. This potentiation drives the expression of critical gluconeogenic enzymes, including PCK1, ultimately increasing hepatic glucose output. The PRMT1‐v2–PGC‐1α regulatory axis is markedly induced by fasting and diabetes, thus representing a key mechanism underlying elevated blood glucose levels in these pathological states [129]. Furthermore, PRMT1 regulates the transcription factor FoxO1 via arginine methylation, thereby controlling hepatic glucose production. Specifically, PRMT1 catalyzes the asymmetric dimethylation of FoxO1 at arginine residues 248 and 250, which impedes phosphorylation at the adjacent serine 253. This alteration enhances the nuclear retention of FoxO1, thereby facilitating cell survival. Moreover, PRMT1 may modulate transcriptional processes dependent on FoxO1, hence influencing the expression of genes associated with gluconeogenesis in the liver [123]. Beyond glucose metabolism, PRMT1 also regulates lipid homeostasis by methylating the CASP8 and FADD‐like apoptosis regulator (CFLAR). Hepatic PRMT1 overexpression inhibits fatty acid oxidation and stimulates de novo lipogenesis, thereby promoting intrahepatic lipid accumulation. This metabolic reprogramming, coupled with enhanced gluconeogenesis, suggests that PRMT1 coordinately drives NAFLD progression by dysregulating both hepatic glucose and lipid metabolism [130]. Thus, PRMT1, specifically the PRMT1V2 isoform, promotes the pathogenesis of NAFLD by methylating key transcriptional regulators such as PGC‐1α and FOXO1, thereby augmenting hepatic gluconeogenesis and exacerbating hyperglycemia. Concurrently, PRMT1 modulates lipid metabolic pathways, further driving hepatic steatosis and disease progression. Consequently, pharmacological targeting of PRMT1 may offer a novel therapeutic avenue for the management of NAFLD and associated metabolic disorders.

##### 5.1.1.4 PRMT1 Plays a Role in NAFLD by Regulating TG Homeostasis

The reduction of PRMT1 in adipocytes reduces fat mass while concurrently activating the AMPK system, resulting in increased lipid droplet size, enhanced lipolysis, and improved mitochondrial lipid catabolism [131]. Conversely, PRMT1 depletion is associated with increased adipose tissue inflammation and ectopic TG accumulation, alterations that can promote insulin resistance [128]. These findings suggest that PRMT1 plays a critical role in the regulation of TG homeostasis. Specifically, Xu et al. [17] demonstrated in both diet‐induced obese mice and obese human subjects that PRMT1 reduces hepatic TG accumulation through upregulation of PGC‐1α expression and enhancement of fatty acid oxidation. In contrast, Chen et al. [130] reported an opposing mechanism wherein PRMT1 mediates CFLAR methylation, facilitating its ubiquitin‐dependent degradation. This degradation relieves the CFLAR‐induced suppression of the JNK pathway, thereby promoting fatty acid synthesis, inhibiting fatty acid oxidation, and exacerbating intrahepatic TG accumulation. Supporting this model, PRMT1 knockdown in methionine‐choline deficient diet‐induced NAFLD models significantly ameliorates hepatic steatosis and reduces TG content. Under physiological conditions, however, PRMT1 overexpression does not alter intrahepatic TG levels but elevates serum TG and fasting glucose, suggesting enhanced VLDL secretion. Mechanistic investigations further revealed that PRMT1 directly interacts with and methylates CFLAR, leading to its degradation, subsequent JNK pathway activation, and consequent hepatic lipid deposition [130]. Therefore, it can be speculated that PRMT1 exerts a dual role in regulating hepatic TG homeostasis in NAFLD. Its function depends on nutritional status, the specific disease model, and the balance among downstream targets, potentially contributing to either amelioration or exacerbation of hepatic steatosis.

##### 5.1.1.5 PRMT1 Contributes to NAFLD Through Regulating Inflammatory Responses

Chronic low‐grade inflammation is a hallmark of obesity, wherein adipocytes act as a major source of proinflammatory cytokines, and their secretory profile plays a central role in driving meta‐inflammation [132]. As inflammatory responses are a major pathogenic driver of NAFLD, their regulation is a crucial therapeutic target [133]. PRMT1 is a key modulator of inflammatory responses, yet it exhibits context ‐ dependent and often paradoxical roles that can either suppress or exacerbate the inflammatory cascade. A primary pathway through which PRMT1 acts is the NF‐κB signaling pathway, which plays a significant role in the transcriptional regulation of genes involved in inflammation and cell survival [134]. For instance, PRMT1 can promote inflammatory reactions by methylating the p65 subunit of NF‐κB, facilitating its association with poly‐ADP‐ribose polymerase 1 and enhancing the transcription of NF‐κB‐dependent genes [135].

In contrast, PRMT1 can also exert anti‐inflammatory effects. Yan et al. [136] used a myeloid‐specific PRMT1 knockout mouse model, demonstrating that loss of PRMT1 resulted in enhanced production of proinflammatory cytokines, reduced survival rates, and increased susceptibility to infections mediated by both DNA and RNA viruses. This anti‐inflammatory function is further evidenced by findings that PRMT1‐mediated asymmetric dimethylation of RelA can inhibit the TNFα/NF‐κB signaling pathway [46]. Moreover, Tikhanovich et al. [137] showed that PRMT1 promotes anti‐inflammatory M2 macrophage polarization by epigenetically regulating PPARγ expression. Consequently, PRMT1 deficiency drives macrophages toward a proinflammatory M1 phenotype, exacerbating inflammatory damage and mortality during infection [137].

Beyond transcriptional regulation, PRMT1 activity in macrophages also controls inflammatory responses by modulating apoptosis. PRMT1 upregulation methylates arginine residues on GAPDH, obstructing its interaction with the proapoptotic protein BAX, thereby inhibiting BAX activation and apoptosis [138]. Additionally, in lipopolysaccharide (LPS)‐induced inflammation models using RAW264.7 macrophages, LPS stimulation downregulates PRMT1 expression and reduces global arginine methylation [139].

Collectively, these findings elucidate the intricate and dualistic function of PRMT1 in inflammation. Future research should investigate the determinants of PRMT1's varied roles, including cell type, inflammatory context, and disease state, as well as its regulatory mechanisms and interactions with other signaling molecules. Such studies are essential to resolve existing discrepancies and may offer novel therapeutic insights.

##### 5.1.1.6 PRMT1 Exerts its Function in NAFLD by Regulating Lipid Metabolism

PRMT1 modulates lipid metabolism in the kidneys, liver, and adipose tissue in a manner that is particular to both the organ and the substrate. It predominantly exerts inhibitory effects in the renal system. In renal tubular epithelial cells, PRMT1 augments the neddylation activity of NEDD4 by methylating the R169 site of UBE2m, thereby facilitating the ubiquitination and degradation of PPARγ, impeding fatty acid oxidation, and ultimately aggravating kidney damage generated by calcium oxalate crystals [140]. PRMT1 expression and function in the liver are contingent upon context. Despite elevated PRMT1 expression in response to a HFD or obesity, SAM deficiency restricted its methylation activity. Currently, more suppression of PRMT1 would diminish HNF‐4α methylation and PGC‐1α transcription, markedly impede fatty acid oxidation, and exacerbate hepatic steatosis. Conversely, overexpression of wild‐type PRMT1 (but not a methyltransferase‐dead mutant) via recombinant adeno‐associated virus reinstates PGC‐1α levels, enhances fatty acid oxidation, and mitigates NAFLD [17]. PRMT1 is capable of methylating Caspase 8 and FADD‐like apoptotic regulator, activating JNK signaling, and facilitating lipid deposition, indicating that its substrate selectivity may fluctuate with clinical situations or dietary models [130]. In adipose tissue, PRMT1 is prominently upregulated in response to a HFD and in human obesity. Adipocyte‐specific PRMT1 deficiency reduces fat mass without affecting total body weight. Mechanistically, PRMT1 knockdown activates the AMPK pathway, leading to enlarged lipid droplets, enhanced lipolysis, and improved mitochondrial lipid catabolism [119]. Furthermore, PRMT1 methylates and activates PGC‐1α, upregulating thermogenic genes like Ucp1 and Cidea to facilitate adaptive thermogenesis. Nonetheless, there remains an absence of direct assessment of fatty acid oxidation in adipose‐specific PRMT1 deletion or activation animals [141].

#### 5.1.2 The Role of PRMT1 in Hepatic Fibrosis

**FIGURE 5:**
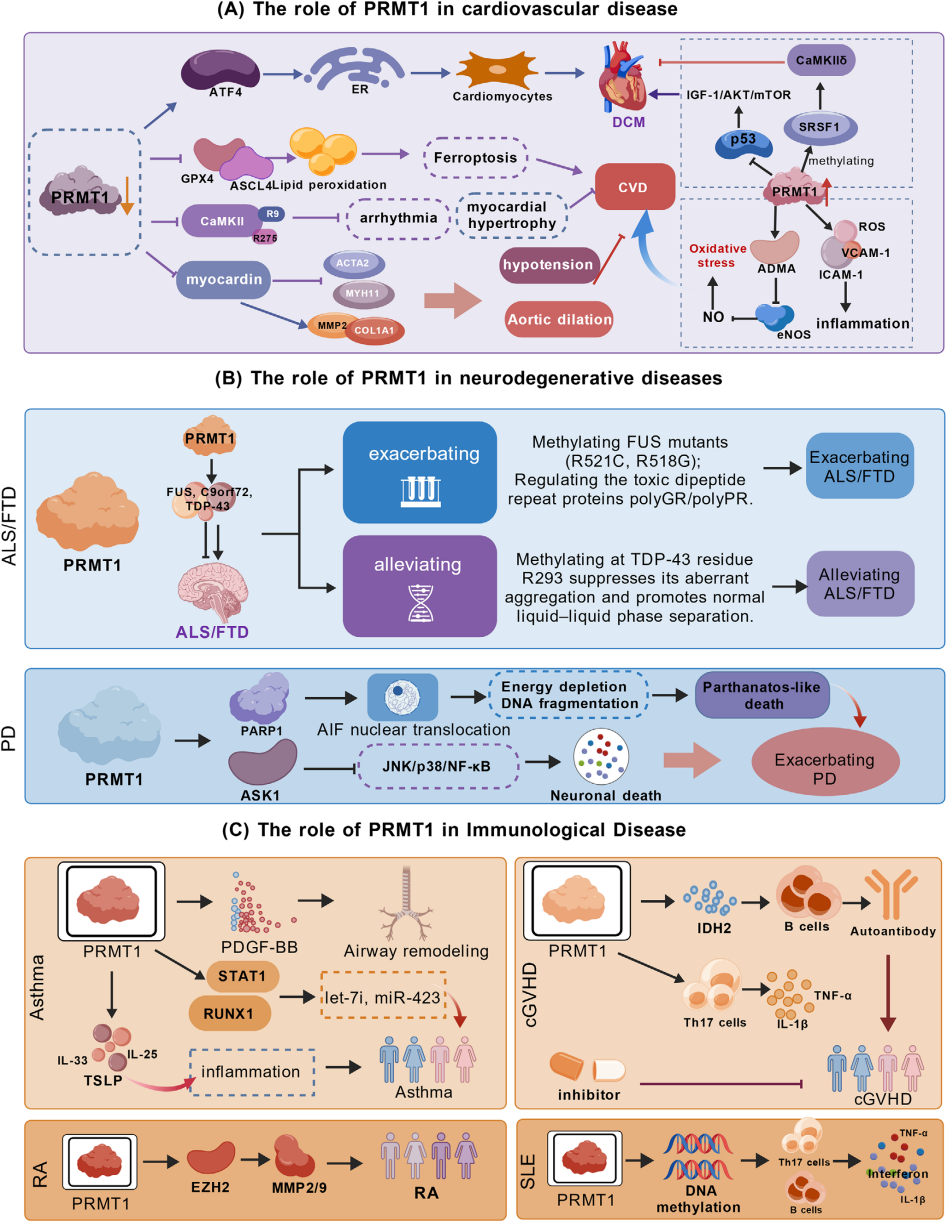
The role of PRMT1 in cardiovascular, neurodegenerative, and immunological diseases. (A) The role of PRMT1 in cardiovascular diseases. PRMT1 affects ferroptosis, endothelial function, myocardial hypertrophy, and the phenotypic transformation of VSMCs in CVD. (B) The role of PRMT1 in neurodegenerative diseases. PRMT1 modulates protein aggregation and neuronal survival, thereby influencing the pathogenesis of NDs. (C) The role of PRMT1 in immunological diseases. PRMT1 regulates the functions of T cells, B cells, and macrophages, thereby influencing immunological diseases.

Hepatic fibrosis represents a reparative fibrogenic reaction ensuing from diverse etiologies of chronic hepatic damage, characterized by the activation of hepatic stellate cells (HSCs) and the excessive deposition of extracellular matrix (ECM). Advanced hepatic fibrosis can progress to severe complications, including cirrhosis, liver failure, and portal hypertension [142]. Inhibition of PRMT1 reduces the activation of HSCs, the primary cell types responsible for synthesizing collagen and other ECM proteins during hepatic fibrogenesis. Thus, PRMT1 activity may be closely linked to the advancement of liver fibrosis in NAFLD. Consequently, inhibiting PRMT1 may present a viable therapeutic approach for addressing hepatic fibrosis associated with NAFLD [143]. Research indicated that PRMT1 expression is heightened in the livers of cirrhotic individuals and that it significantly contributes to hepatic fibrogenesis. Considering liver fibrosis, PRMT1 is significantly elevated in both hepatocytes and HSCs in the fibrotic liver tissue. A specific PRMT1 inhibitor, such PT1001B, significantly reduces PRMT1 activity, therefore mitigating hepatic fibrosis in mice. The targeted deletion of PRMT1 in HSCs reduces HSC activation and hepatic fibrosis in the TAA‐induced fibrotic model. The ablation of PRMT1 in HSCs markedly inhibits the proinflammatory NF‐κB and profibrogenic TGF‐β signaling pathways, while also reducing the production of profibrogenic mediators in murine livers [143]. The results indicate that inhibiting PRMT1 could serve as an effective therapeutic approach for managing liver fibrosis by reducing HSC activation. This is a crucial factor in the formulation of therapies designed to impede the advancement of liver fibrosis and potentially reverse the disease (in Figure 5).

#### 5.1.3 The Role of PRMT1 in HCC

PRMTs significantly influence tumorigenesis and cancer progression by modulating critical processes such as RNA splicing and cellular proliferation [144]. Among them, PRMT1 is overexpressed in various cancers, including HCC. In HCC, elevated PRMT1 expression is inversely correlated with the infiltration of CD8^+^ T cells and macrophages within the tumor microenvironment, suggesting an immunosuppressive role. Moreover, high PRMT1 expression is negatively correlated with patient prognosis, indicating its association with inferior therapeutic outcomes and diminished survival rates [48]. PRMT1 exhibits aberrant expression in liver cancer patient samples, where its levels are correlated with HCC progression, modulation of the tumor immune microenvironment, and dysregulation of fatty acid metabolism [145]. Specifically, PRMT1 expression is associated with altered immune cell infiltration, particularly in immunotherapy contexts, involving exhausted CD8^+^ T cells, B cells, and monocytes/macrophages. Moreover, in PRMT1‐knockdown HCC cells, cellular viability, migration, invasion, and the expression of genes associated with fatty acid metabolism are diminished. Genes coexpressed with PRMT1 participate in fatty acid metabolism and are prevalent in fatty liver disease and drug‐induced hepatic disorders. These findings establish a foundation for additional research and underscore possible clinical treatment targets for HCC [146].

Recent study advancements have underscored the function of PRMT1 in augmenting the activity of PHGDH via methylation, thereby facilitating serine production and demonstrating tumor‐promoting effects on HCC in both in vitro and in vivo studies. Moreover, increased expression levels of PRMT1 are significantly correlated with unfavorable prognosis in HCC patients, indicating its potential as a predictive biomarker for treatment efficacy and survival outcomes [145]. The E3 ubiquitin ligase FBXO7 interacts with PRMT1, facilitating its ubiquitination at lysine 37, which leads to the proteasomal degradation of PRMT1, hence limiting serine production and the proliferation of HCC cells. FBXO7 expression is diminished in human HCC tissue, and its expression level exhibits a negative correlation with the methylation levels of PRMT1 and PHGDH, presenting novel molecular pathways and possible therapeutic targets for cancer treatment [147].

Alcohol consumption is a significant risk factor for HCC. PRMT1 serves a protective function against alcohol‐induced hepatotoxicity by regulating hepatocyte proliferation and oxidative stress. PRMT1 knockout mice exhibit a higher incidence of alcohol‐promoted liver tumors, which is associated with enhanced hepatic proliferation, activation of the WNT/β‐catenin signaling pathway, and intensified inflammatory responses. Mechanistically, PRMT1 modulates this process by regulating intracellular NO levels. PRMT1 catalyzes the production of ADMA, a competitive inhibitor of NOS. Consequently, PRMT1 deficiency leads to reduced ADMA, resulting in excessive NO generation, oxidative stress, and inflammatory responses that drive liver injury and carcinogenesis. The critical role of this pathway is demonstrated by the fact that pharmacological inhibition of inducible NOS attenuates excessive NO production, thereby reducing hepatocyte apoptosis and inflammation. This intervention consequently suppresses the activation of the WNT/β‐catenin signaling pathway and inhibits tumor proliferation. Therefore, PRMT1 acts as a critical protective agent against alcohol‐induced hepatic damage, inflammation, and HCC progression [148]. Furthermore, PRMT1 substantially mitigates alcohol‐induced oxidative stress, inflammation, and cellular apoptosis by suppressing inducible NOS. This protective action ultimately reduces the incidence of HCC in models combining diethylnitrosamine initiation with prolonged alcohol consumption. Importantly, this tumor‐suppressive function of PRMT1 appears specific to alcohol‐associated carcinogenesis. The underlying mechanism involves PRMT1‐mediated regulation of key pathways governing cellular proliferation, the expression of inflammatory markers, and the production of antioxidant proteins. Disruption of this PRMT1‐regulated network is therefore implicated in the pathogenesis of alcohol‐induced HCC. Collectively, these findings establish PRMT1 as a key protective factor and provide a compelling rationale for exploring its therapeutic potential in preventing and treating ethanol‐associated HCC [149].

Thymidine kinase 1 (TK1) has been recognized as a pivotal factor in the advancement of HCC, facilitating carcinogenesis via its enzymatic function and by influencing metabolic reprogramming. TK1 specifically augments glycolysis and glycolysis‐associated malignancy in HCC through the upregulation of PRMT1. Mechanistically, TK1 stabilizes PRMT1 by directly binding to it and disrupting its interaction with tripartite‐motif‐containing 48, hence preventing the ubiquitination‐mediated degradation of PRMT1. This connection is crucial as it enables the further methylation of metabolic enzymes, including phosphofructokinase PFKFB3, hence promoting the glycolytic pathway in HCC. Moreover, investigations of clinical samples have revealed a robust relationship between the expression levels of TK1 and PRMT1 and tumor volume, microvascular invasion, and tumor stage in patients with HCC. Significantly, increased expression levels of TK1 and PRMT1 are coincident with unfavorable prognosis in HCC patients, indicating their potential as therapeutic targets [150]. Furthermore, PRMT1‐mediated methylation of p110α suppresses stress granule formation, thereby reducing sorafenib resistance in HCC. Based on this mechanism, the developed R612F nanoparticles effectively enhance therapeutic efficacy [151]. In conclusion, the many modes of action of PRMT1 in HCC make it an attractive therapeutic target. Inhibitors of PRMT1 or targeted medicines that adjust its regulatory processes may offer innovative approaches for the treatment of HCC. Subsequent research will investigate the precise mechanisms of PRMT1 in HCC and evaluate its efficacy and safety as a therapeutic target.

### 5.2 The Role of PRMT1 in CVD

In recent years, increasing evidence has shown that the expression and function of PRMT1 are tightly associated with the physiological homeostasis and pathological processes of the cardiovascular system. PRMT1 has a multifaceted and significant involvement in various critical aspects of cardiovascular disorders, including the survival and hypertrophy of cardiomyocytes, the proliferation of vascular smooth muscle cells (VSMCs), and the failure of endothelial function. This chapter will methodically examine the precise role and molecular mechanism of PRMT1 in a cardiac‐specific deletion model, myocardial hypertrophy, VSMC function, and endothelial dysfunction.

#### 5.2.1 Cardiac‐Specific PRMT1 Ablation

To elucidate the essential function of PRMT1 in cardiac physiology, a cardiomyocyte‐specific PRMT1‐knockout mouse model was generated. Murata et al. [152] reported that these knockout mice developed dilated cardiomyopathy during juvenile stages, which was characterized by disorganized myocardial structure, thinning ventricular walls, chamber dilation, and significant fibrosis. Similarly, Pyun et al. [153] demonstrated that adult mice with cardiomyocyte‐specific PRMT1 ablation spontaneously developed left‐ventricular dilation, a markedly reduced ejection fraction, myocardial hypertrophy, and fibrosis. These mice also exhibited characteristic electrophysiological abnormalities, including QT prolongation and sinus bradycardia [153]. Collectively, these findings demonstrate that PRMT1 deficiency alone is sufficient to induce severe structural and electrophysiological abnormalities in the heart, even in the absence of external stress. Therefore, PRMT1 is indispensable for maintaining normal cardiac architecture and function throughout postnatal life.

PRMT1 diminishes the protein stability of the transcription factor activating transcription factor 4 (ATF4) at the arginine 239 location through methylation, hence obstructing the ER stress pathway governed by ATF4/CHOP and mitigating cardiomyocyte death. The cardiac‐specific deletion of PRMT1 amplifies the ER stress response, resulting in premature postpartum cardiomyocyte apoptosis and impaired heart function [154]. Furthermore, PRMT1‐facilitated ATF4 methylation can mitigate ER stress, hence reducing doxorubicin‐induced cardiotoxicity [155]. In the congenital heart disease model generated by environmental pollutants (such as formaldehyde), PRMT1 was modulated by lncRNA 91,234.1, which influenced the expression of its downstream target ASCL4, subsequently regulating the expression of glutathione peroxidase 4 (GPX4). The PRMT1‐mediated asymmetric dimethylation of the histone H4R3 region alters GPX4 expression, resulting in lipid peroxide buildup and the induction of ferroptosis in cardiomyocytes [156]. In conclusion, aberrant expression or malfunction of PRMT1 is significantly linked to numerous cardiovascular illnesses, positioning it as a prospective therapeutic target for future treatments.

#### 5.2.2 The Role of PRMT1 in Myocardial Hypertrophy

Myocardial hypertrophy represents an initial compensatory response of the heart to hemodynamic stress, such as pressure overload from hypertension or aortic stenosis, and neurohumoral activation. However, when sustained, this adaptive remodeling transitions into a pathological state that progresses to ventricular dysfunction and heart failure [157]. PRMT1 can reduce calcium/calmodulin‐dependent protein kinase II (CaMKII) activity by methylating arginine residues (e.g., R9 and R275) of CaMKII, hence inhibiting arrhythmia and cardiac hypertrophy resulting from excessive CaMKII activation. Moreover, PRMT1 modulates the function of the slow delayed rectifier potassium channel by enhancing the interaction of the KCNQ1 subunit with the membrane lipid PIP2 through methylation, thus preserving the channel's open state and facilitating the proper repolarization of the cardiac action potential. The ablation or inhibition of PRMT1 will diminish the functionality of IKs, extend the action potential length, and elevate the risk of arrhythmia [158]. In an isoproterenol‐induced animal model of myocardial hypertrophy, PRMT1 expression was significantly downregulated. This loss of PRMT1 function exacerbated cardiomyocyte hypertrophy, fibrosis, and apoptosis, leading to a decline in cardiac function. Conversely, PRMT1 overexpression attenuated these pathological remodeling processes, confirming its potent antihypertrophic role. Mechanistically, PRMT1 interacts with and methylates the RNA splicing factor SRSF1, which inhibits its phosphorylation. This PRMT1‐mediated regulation of SRSF1 alters the alternative splicing of CaMKIIδ, promoting the expression of the A and B isoforms while suppressing the C isoform. The resultant shift in CaMKIIδ isoform expression ultimately attenuates the development of myocardial hypertrophy [159]. PRMT1 has a role in cardiac development and EMT via modulating p53 stability and the alternative splicing of MDM4, hence influencing the EMT of epicardial cells. This process regulates the formation of cardiac fibroblasts, coronary VSMCs, and pericytes, ultimately affecting ventricular morphogenesis and the development of coronary arteries [160]. PRMT1 can directly bind to p53 and impede its transcriptional activity in an enzyme‐dependent manner, leading to a reduction in the expression levels of several critical downstream targets of the p53 pathway. p53 can upregulate target genes such as PTEN, TSC2, and SESN1/2, while inhibiting IGF‐1/AKT/mTOR signaling, therefore diminishing cardiac hypertrophy [161]. Consequently, it is hypothesized that PRMT1 may suppress the expression of p53 and its downstream targets, so facilitating myocardial hypertrophy.

#### 5.2.3 The Role of PRMT1 in VSMCs

The abnormal proliferation and migration of VSMCs are fundamental pathogenic mechanisms in vascular disorders, including atherosclerosis, hypertension, and vascular restenosis [162]. PRMT1 plays a critical protective role in VSMCs, and its deficiency can lead to aortic dissection, elastic fiber degeneration, and apoptosis. PRMT1‐deficient mice exhibit severe hypotension and aortic dilatation, reflecting this loss of vascular integrity. The underlying mechanism involves a PRMT1 deficiency‐induced phenotypic switch in VSMCs, characterized by a significant downregulation of contractile genes, including ACTA2, MYH11, and CNN1, and a concurrent upregulation of synthetic genes, such as COL1A1, COL3A1, and matrix metalloproteinase 2 (MMP2). This transition from a contractile to a synthetic phenotype is driven, in part, by the suppression of the key contractile regulator myocardin. PRMT1 promotes myocardin transcription by facilitating the asymmetric dimethylation of H4R3me2a at the myocardin promoter. Consequently, PRMT1 deficiency reduces activating histone marks (e.g., H3K9ac) and increases repressive marks (e.g., H3K27me3) at this locus, thereby suppressing myocardin expression and initiating the pathological phenotypic switch [163].

#### 5.2.4 The Role of PRMT1 in Endothelial Dysfunction

Endothelial dysfunction serves as the initial catalyst for nearly all cardiovascular disorders. The primary characteristics include reduced bioavailability of the arterial dilation factor NO and the development of proinflammatory and prothrombotic conditions [164]. PRMT1 is recognized for its negative regulatory function in this process, primarily via producing the endogenous NOS (eNOS) inhibitor, ADMA. eNOS is an enzyme that enhances cardiovascular health through many mechanisms, while ADMA, as a competitive inhibitor of eNOS, diminishes NO generation, resulting in compromised endothelial function [165]. The presence of many cardiovascular risk factors, including hyperlipidemia and oxidative stress, will elevate the expression and activity of PRMT1 in endothelial cells, resulting in increased synthesis of ADMA [166, 167]. Elevated levels of ADMA directly inhibit eNOS activity. This inhibition leads to reduced NO production, impaired endothelium‐dependent vasodilation, and consequently, elevated blood pressure. Furthermore, eNOS inhibition by ADMA can induce a state of eNOS uncoupling. In this uncoupled state, eNOS shifts from producing NO to generating substantial amounts of superoxide anions. This overproduction of superoxide exacerbates oxidative stress, which in turn further impairs endothelial function, creating a vicious cycle of vascular dysfunction [168]. PRMT1 facilitates damage in LPS‐induced endothelial dysfunction by augmenting protein arginine methylation, elevating ADMA levels, suppressing NO generation, advancing cell senescence, and activating inflammatory pathways [169]. Besides producing ADMA, PRMT1 may influence endothelial function through other mechanisms. The stimulation by LPS or elevated salt levels upregulates PRMT1 expression. This, in turn, enhances the formation of reactive oxygen species and upregulates inflammatory factors such as VCAM‐1 and ICAM‐1, ultimately promoting endothelial cell activation and dysfunction [170]. PRMT1 may modulate proteins associated with the VEGF signaling cascade through methylation, contribute to endothelial cell migration, proliferation, and lumen creation, and influence vascular repair and remodeling [171].

In conclusion, the overactivation of PRMT1 in endothelial cells primarily results in a decrease in NO, increased oxidative stress, and exacerbated inflammation through both ADMA‐dependent and independent mechanisms, thus forming the pathological foundation of endothelial dysfunction. The targeted suppression of PRMT1 or the enhancement of ADMA breakdown, such as through the DDAH enzyme, has emerged as a significant research focus for enhancing endothelial function and preventing and treating cardiovascular disorders.

### 5.3 The Role of PRMT1 in NDs

NDs, including Alzheimer's disease (AD), PD, amyotrophic lateral sclerosis (ALS), and Huntington's disease (HD), represent a category of debilitating disorders marked by the gradual degeneration of neurons in the central nervous system [172, 173, 174]. Despite differing clinical manifestations, an increasing body of evidence suggests that many illnesses share fundamental molecular processes, such as protein misfolding and aggregation, mitochondrial dysfunction, impaired DNA damage repair, and disrupted RNA metabolism. PTMs are essential regulators of biological activities. Arginine methylation, performed by PRMT1, has emerged as a pivotal PTM that regulates neuronal survival and function; dysregulation of this alteration contributes to the initiation and progression of various NDs.

#### 5.3.1 Alzheimer's Disease

The characteristic clinical hallmarks of AD include the aberrant aggregation of extracellular amyloid β (Aβ) peptides in neurons, resulting in the formation of senile plaques. The tau protein in neuronal cells of the brain becomes twisted into bundles due to excessive phosphorylation, resulting in the formation of neurofibrillary tangles [175]. Genetic and epigenetic pathways have been shown to significantly influence the pathogenesis of AD. PRMTs are linked to the disease and play a role in regulating multiple signaling pathways, ultimately resulting in Aβ buildup, tau phosphorylation, neuroinflammation, and cell death [176, 177]. However, current research mostly highlights the essential roles of PRMT4, PRMT5, and PRMT8 in AD, with no studies specifically clarifying the importance of PRMT1 in this regard.

#### 5.3.2 ALS and Frontotemporal Dementia

ALS and frontotemporal dementia (FTD) are NDs with overlapping clinical and pathological features. A core pathological mechanism involves the mislocalization and aggregation of RNA‐binding proteins such as tyrosyl‐DNA phosphodiesterase (TDP)‐43 and FUS in the cytoplasm, although not all cases exhibit this hallmark [178]. PRMT1 directly interacts with and asymmetrically dimethylates the ALS‐associated protein FUS (fused in sarcoma), a modification that regulates its nucleocytoplasmic transport, aggregation propensity, and cytotoxicity. In ALS patients, FUS mutations (such as R521C and R518G) can still bind to PRMT1 and be methylated, but mutant FUS is more likely to form cytoplasmic inclusions and detain PRMT1 in stress granules, resulting in the loss of its nuclear function, which further aggravates the cytoplasmic aggregation and neurotoxicity of FUS [179]. In a Drosophila model, the knockdown of the PRMT1 homolog DART1 intensified neurotoxicity induced by FUS overexpression, indicating that PRMT1 had a neuroprotective function [180]. The C9orf72 gene repeat expansion model, the predominant genetic contributor to ALS/FTD, implicates PRMT1‐mediated arginine methylation in the modulation of hazardous dipeptide repeat proteins, including polyGR and polyPR. Inhibiting PRMT1 activity, for instance through the use of the inhibitor MS023, can mitigate polyGR/polyPR‐induced neuronal toxicity, enhance neuronal survival, and preserve axonal integrity, indicating that PRMT1 exerts a pathogenic influence in C9orf72‐related ALS/FTD [181]. TDP‐43 is a crucial pathogenic protein in ALS/FTD, with its aberrant phosphorylation and aggregation serving as a disease hallmark. PRMT1 prevents the aberrant aggregation of TDP‐43 and enhances its normal liquid‐liquid phase separation function by methylating the R293 site. Conversely, p38α‐mediated phosphorylation (notably at S292 and S409/410) inhibits the methylation of R293, hence facilitating TDP‐43 aggregation and toxicity. Consequently, PRMT1 and p38α exert opposing influences on TDP‐43 protein lesions, and the activation of PRMT1 may possess therapeutic potential [182]. In motor neurons obtained from ALS patients, the nucleo‐cytoplasmic localization of PRMT1 is aberrant, and its compromised function is intimately associated with the atypical aggregation of proteins such as FUS and TDP‐43. Research indicates that the expression of PRMT1 is modulated by the RNA‐binding protein RALY. RALY downregulation diminishes PRMT1 levels, thereby influencing FUS methylation and localization, indicating that PRMT1 is crucial to the regulatory network of RNA metabolism. Under oxidative stress, PRMT1 and FUS colocalize in stress granules, and their methylation activity influences the stability and removal of these granules. The loss of PRMT1 results in the sustained presence of FUS‐positive stress granules, subsequently leading to neurite shrinkage and neuronal dysfunction [183].

#### 5.3.3 Parkinson's Disease

PD is defined by the degeneration of dopaminergic neurons in the midbrain substantia nigra and the development of Lewy bodies due to the aggregation of α‐synuclein [184]. In the neurotoxic models of MPP^+^ and rotenone, the expression and activity of PRMT1 were markedly elevated. The overexpression of PRMT1 may exacerbate the apoptosis of dopaminergic neurons, whereas gene knockout or heterozygous deletion (PRMT±) can substantially diminish cell death and save the substantia nigra neurons in mice [18]. Furthermore, PRMT1 facilitates the nuclear translocation of apoptosis‐inducing factor (AIF) by augmenting PARP1 overactivation, resulting in energy depletion and DNA breakage, thus promoting parthanatos‐like cell death, as evidenced in both in vivo and in vitro PD models [185]. Besides PARP1‐mediated AIF regulation, PRMT1 may influence the apoptosis of dopaminergic cells by additional pathways, including the methylation and modulation of apoptosis signal‐regulating kinase 1 (ASK1). PRMT1 facilitates arginine methylation at positions 78/80 of ASK1, impedes the ASK1–TRAF2 connection, and promotes its association with thioredoxin, therefore downregulating the ASK1/JNK and ASK1/p38/NF‐κB pathways, thereby influencing the threshold of neuronal inflammation and death [186]. The latest research revealed that in MPP^+^‐treated dopaminergic neurons, PRMT1 augmented EZH2 stability by obstructing EZH2–T311 phosphorylation, which subsequently suppressed the expression of suppressor of cytokine signaling 3 (SOCS3), leading to a reduction in cell survival rate and an elevation in death. Inhibiting PRMT1 or reinstating SOCS3 can mitigate this detrimental effect [187].

#### 5.3.4 Huntington's Disease

HD is caused by an aberrant expansion of CAG trinucleotide repeats in the huntingtin (HTT) gene. The resulting mutant HTT protein forms neuronal aggregates and induces widespread transcriptional dysregulation [188]. The role of PRMT1 in HD pathogenesis has been less extensively investigated compared with other PRMTs, such as PRMT4 and PRMT6. Nonetheless, emerging evidence suggests a potential regulatory function for PRMT1 in this disorder. PRMT1 has been shown to interact with the HTT protein, implying a potential role in its PTM via arginine methylation. Although direct methylation of HTT by PRMT1 has not yet been confirmed, PRMT1, as the principal type I methyltransferase, could significantly modulate HTT function and toxicity [189].

### 5.4 The Role of PRMT1 in Immunological Disease

The ability of the immune system to maintain homeostasis and respond effectively to pathogens depends on a complex and precise network of cells and molecules. PTMs are key regulatory mechanisms that finely regulate the activity of restriction factors and determine the strength and duration of innate immune responses, thereby contributing to the maintenance of immune homeostasis [190]. Arginine methylation, catalyzed by the PRMT family, is an emerging PTM that regulates multiple aspects of immune cell function and is increasingly associated with innate and adaptive immunity [191].

#### 5.4.1 Molecular Mechanism of PRMT1 in Immune Regulation

PRMT1 catalyzes the asymmetric dimethylation of H4R3me2a, an epigenetic mark associated with open chromatin. This modification facilitates chromatin relaxation and enhances the recruitment of transcription factors and coactivators, thereby promoting the transcription of downstream genes [15]. PRMT1 has been shown to play a critical role in immune cells, including T lymphocytes, B lymphocytes, and macrophages. For instance, it is required for cytokine production by T helper cells [192]. PRMT1 affects the expression of cytokines such as IFN‐γ and IL‐4 in Th1 and Th2 cells. It promotes the interaction between NIP45 and NFAT by methylating NIP45, thereby activating the transcription of related genes [193]. PRMT1 methylates cyclin‐dependent kinase 4 and Igα, regulating B cell proliferation, differentiation, and antibody production. PRMT1 deficiency leads to humoral immunodeficiency [55, 194]. In addition, PRMT1 plays a critical role in the production of IL‐6 in macrophages [195]. Fan et al. found that PRMT1 can directly bind to CIITA and methylate CIITA, thereby accelerating the degradation of CIITA, shortening its half‐life, and ultimately weakening the activation of CIITA on the MHC II promoter and inhibiting MHC II expression. IFN‐γ is the main inducer of MHC II expression, and its mechanism may be to downregulate the mRNA and protein expression of PRMT1 and reduce its binding to the MHC II promoter [196].

The NF‐κB signaling pathway is a central regulator of inflammation and immunity. PRMT1 serves as a key negative regulator of this pathway by directly methylating the p65 (RelA) subunit at the R30 site. This methylation event inhibits p65's DNA‐binding capacity, thereby suppressing the expression of NF‐κB‐mediated inflammatory genes. This regulatory mechanism is critical for the resolution phase of the inflammatory response, as it helps prevent excessive tissue damage [46]. In a separate immunomodulatory pathway, PRMT1 methylates the DNA sensor cyclic GMP‐AMP synthase (cGAS) at a conserved N‐terminal arginine residue (R133). This methylation prevents cGAS dimerization and membrane localization, leading to inhibition of the cGAS–STING pathway and a blunted type I interferon response. This process is exploited in the tumor microenvironment to facilitate immune escape. Consequently, pharmacological inhibition of PRMT1 can enhance the antitumor immune response and synergize with anti‐PD‐1 immunotherapy [109].

In summary, PRMT1 is a central regulator of immune response, inflammation regulation, and tumor immune escape, achieved by methylating diverse nonhistone substrates and finely regulating key immune signaling pathways such as cGAS–STING, NF‐κB, and MHC‐II. Studying its mechanism not only deepens the understanding of immune regulation but also provides novel targets and strategies for treating immune diseases.

#### 5.4.2 The Role of PRMT1 in Immunological Diseases

Dysregulation of PRMT1 activity and expression is significantly associated with the pathogenesis of various immune‐mediated diseases, including rheumatoid arthritis (RA), systemic lupus erythematosus (SLE), asthma, and chronic graft‐versus‐host disease (cGVHD). PRMT1 is essential and has been recognized as a potential therapeutic target for immune disorders, despite the challenges involved.

##### 5.4.2.1 Rheumatoid Arthritis

RA is a prevalent immune‐mediated inflammatory disease, classified as a chronic autoimmune disorder marked by synovial inflammation and joint destruction [197]. Expression levels of PRMT1 in the synovial tissue of patients with RA were significantly elevated compared with the normal control group. The heightened expression is predominantly noted in fibroblast‐like synoviocytes (FLS), which are crucial in joint degradation and the progression of inflammation in RA. PRMT1 promotes the migration, invasion, and antiapoptotic characteristics of RA–FLS through the upregulation of MMPs, specifically MMP‐2 and MMP‐9. MMPs degrade articular cartilage and bone tissue, leading to the destruction of joint structure. Liu et al. demonstrated that the effect of PRMT1 was partially mediated by the upregulation of EZH2. EZH2 functions as a histone methyltransferase, influencing both cell proliferation and the inflammatory response. PRMT1 may activate EZH2 through methylation modification, leading to an increase in MMP expression and the pathogenicity of synovial cells [198]. The inhibition of PRMT1 is regarded as having therapeutic potential due to its proinflammatory and prodestructive roles in RA. The impact of PRMT1 inhibitors in various inflammatory models, including TC‐E 5003, which inhibits TLR4‐mediated NF‐κB and c‐Jun activation, consequently diminishing the inflammatory response [199]. PRMT1 contributes to the progression of RA by facilitating synovial cell proliferation, inhibiting apoptosis, increasing invasiveness, and upregulating MMP expression. The mechanism includes downstream molecules like EZH2, indicating that the PRMT1/EZH2 axis could serve as a novel target for RA treatment.

##### 5.4.2.2 Systemic Lupus Erythematosus

SLE is a multifaceted systemic autoimmune disorder marked by the generation of autoantibodies, a type I interferon signature, and a breakdown of self‐tolerance [200]. PRMT1 regulates gene expression through the catalysis of arginine methylation on histones. The recruitment of PRMT1 represents a preliminary step in the regulation of gene transcription by nuclear hormones. In patients with SLE, alterations in DNA methylation patterns are significantly associated with the disease's onset and progression. Additionally, PRMT1‐mediated histone methylation may influence these DNA methylation patterns, subsequently regulating immune cell function and differentiation. Alterations in PRMT1 activity could influence the functionality of T cells and B cells. Changes in DNA methylation patterns in T cells can result in abnormal gene expression, leading to the transformation of normal antigen‐specific T cells into autoreactive T cells. Furthermore, PRMT1‐mediated arginine methylation may influence B cell activation and autoantibody production, thereby worsening the immunopathological response in SLE [201]. The mechanism by which PRMT1 operates in SLE is not yet fully understood. Alterations in the expression or activity of this crucial enzyme in epigenetic regulation may correlate with the activity of SLE. The methylation modification mediated by PRMT1 may affect inflammatory pathways, such as the interferon signaling pathway, thus influencing the inflammatory response and tissue damage related to SLE [202]. Considering the regulatory function of PRMT1 in SLE, it is anticipated to serve as a viable therapeutic target.

##### 5.4.2.3 Asthma

Asthma is characterized by airway hyperresponsiveness and chronic airway inflammation. Its pathogenesis is primarily driven by allergic reactions mediated by inflammatory cells such as mast cells and eosinophils, leading to airway constriction and reversible airflow limitation [203]. PRMT1 expression is significantly upregulated in the airway smooth muscle cells (ASMCs) of asthmatic patients, and its levels correlate with the extent of airway remodeling. Airway remodeling, a key pathological feature of asthma, involves thickening of the airway wall, proliferation and migration of ASMCs, and deposition of ECM components such as collagen and fibronectin. PRMT1 enhances the proliferation and migration of ASMCs by modulating the platelet‐derived growth factor BB signaling pathway, thereby promoting airway remodeling and exacerbating disease progression [19]. PRMT1 influences the processing of miRNAs associated with asthma in lung epithelial cells. PRMT1 associates with STAT1 or RUNX1, functioning as a coactivator to enhance the transcription of pri‐miRNA, which subsequently influences the expression of mature miRNA. PRMT1 has the capacity to enhance the transcription of miRNAs, including let‐7i and miR‐423. Abnormal expression of these miRNAs in asthma patients may be associated with the pathogenesis of the condition [204]. PRMT1 is involved in the inflammatory response associated with asthma. It promotes the production of epithelial cell‐derived cytokines, including TSLP, IL‐25, and IL‐33, which are crucial in the immune regulation and inflammatory response associated with asthma [205]. In the allergic asthma model, heightened PRMT1 expression correlated with eosinophil infiltration and increased levels of TH2‐type cytokines, including IL‐4, IL‐5, and IL‐13 [206]. The role of PRMT1 in asthma encompasses various aspects, including airway remodeling, miRNA processing, and the inflammatory response. The mechanism is intricate and varied, offering novel potential targets for asthma treatment.

##### 5.4.2.4 Chronic Graft‐Versus‐Host Disease

cGVHD represents a prevalent immune‐mediated complication following allogeneic hematopoietic stem cell transplantation. This condition is marked by ongoing dysregulation of the immune system and an inflammatory response, resulting in fibrosis and dysfunction of organs. The pathogenesis includes the immune‐mediated assault of donor T cells on host tissues, dysregulated activation of B cells leading to autoantibody production, cytokine imbalance, and an inflammatory response facilitated by immune cells, including macrophages [207]. A significant upregulation of PRMT1 in CD4^+^ T cells and B cells from human cGVHD patients, as well as in two distinct mouse models of the disease (scleroderma‐like and lupus‐like). In these animal models, treatment with a selective PRMT1 inhibitor markedly prevented cGVHD onset, as evidenced by prolonged survival, reduced clinical scores, and attenuated organ damage, including kidney pathology. Mechanistically, PRMT1 inhibition significantly reduced the population of pathogenic Th17 cells, which are key drivers of the inflammation and tissue fibrosis characteristic of cGVHD. Furthermore, the inhibitor diminished the number of germinal center B cells and plasma cells, leading to a reduction in autoantibody production—a critical process in controlling the autoimmune component of cGVHD. Importantly, PRMT1 inhibition did not compromise regulatory T cell function, thereby preserving a crucial mechanism for maintaining posttransplant immune tolerance. At a molecular level, PRMT1 was found to enhance the activity of the metabolic enzyme isocitrate dehydrogenase 2 (IDH2) through methylation. This increase in IDH2 activity promotes B cell proliferation and antibody secretion, thereby exacerbating cGVHD pathology [208]. PRMT1 serves as a crucial regulatory factor in the pathogenesis of cGVHD, influencing the disease's development through its effects on T cell and B cell function. PRMT1 represents a potential target for the prevention and treatment of cGVHD. The role of PRMT1 in cardiovascular, neurodegenerative, and immune diseases is shown in Figure 5.

## 6 Prospect of PRMT1 Inhibitors

**FIGURE 6:**
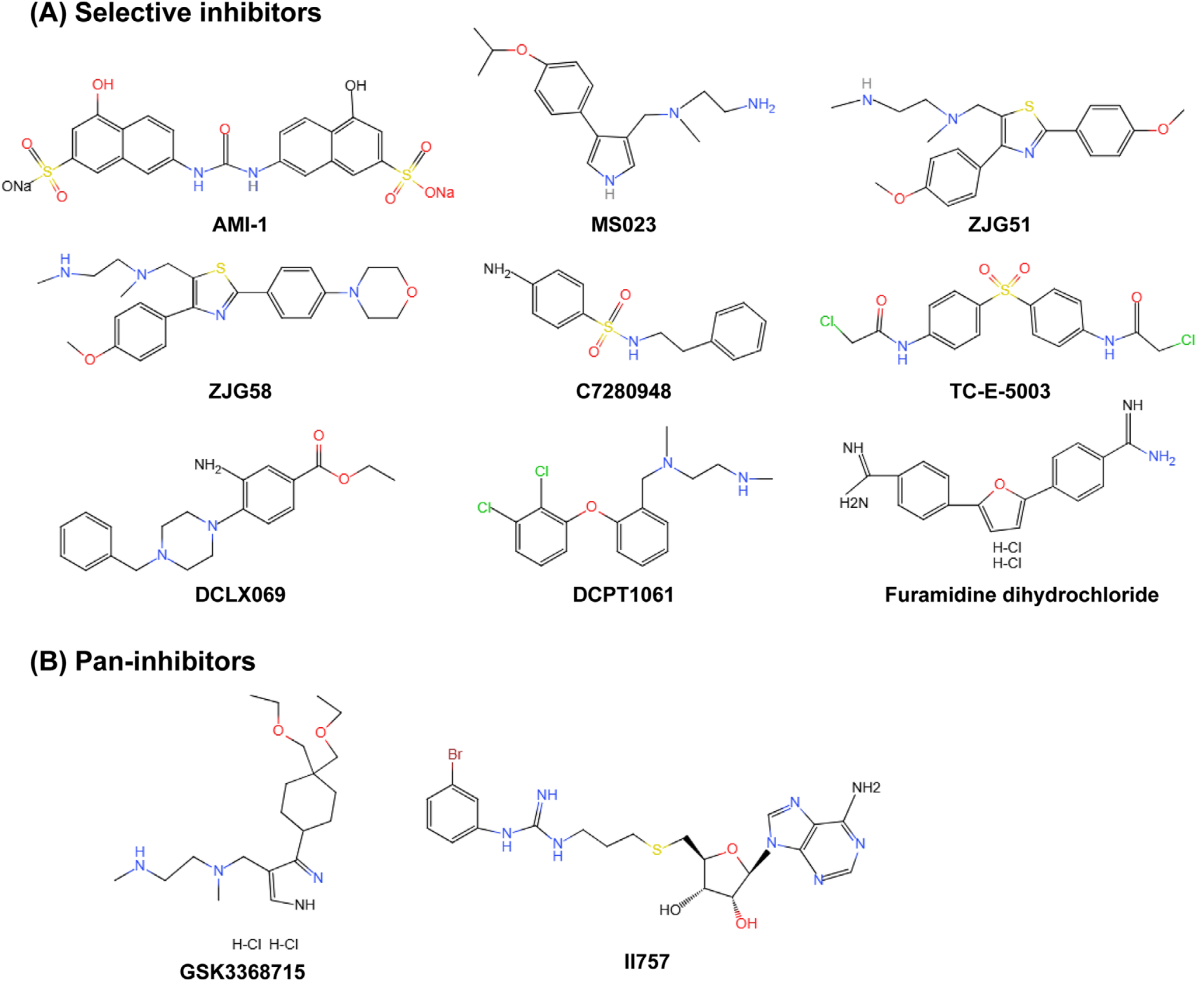
The chemical structure of PRMT1 inhibitors. (A) The chemical structure of selective inhibitors of PRMT1. (B) The chemical structure of pan‐inhibitors of PRMT1. *Note*: All inhibitor structures were drawn using the InDraw website (https://indrawforweb.integle.com/).

The critical involvement of PRMT1 in a spectrum of diseases, as outlined in this review, has spurred significant interest in the development of PRMT1 inhibitors. A summary of these inhibitors, including their detailed characteristics, is provided in Table 2, and their chemical structures are illustrated in Figure 6.

**TABLE 2:** PRMT1 inhibitors.

Classification	PRMT1 inhibitor	Mechanism	IC50	Highest phase	References
Selective inhibitors	AMI‐1	Antioxidant	8.8 µM	Preclinical	[209, 210]
	MS023	Substrate competition	30 nM	Preclinical	[211, 212]
	ZJG51	Engagement with the substrate‐binding domain	11.8 µM	Preclinical	[213]
	ZJG58	Engagement with the substrate‐binding domain	7.1 µM	Preclinical	[213]
	C7280948	Engagement with the substrate‐binding domain	12.8 µM	Preclinical	[214]
	TC‐E‐5003	—	1.5 µM	Preclinical	[99]
	DCLX069	SAM competition	17.9 µM	Preclinical	[57]
	DCPT1061	Regulating the activity of CD8+ T cells; methylation of SRSF1	There is a need for further exploration.	Preclinical	[28, 58]
	Furamidine dihydrochloride (DB75 dihydrochloride)	TDP‐1 competition	9.4 µM	Preclinical	[217]
Pan‐inhibitors	CTS‐2190	—	—	Phase I/II	[20]
	GSK3368715 (EPZ019997)	Substrate competition	3.1 nM	Phase I (termination)	[220]
	II757	SAM competition	16.4 nM	Preclinical	[223]

### 6.1 Selective Inhibitors

#### 6.1.1 Arginine Methylation Inhibitor‐1

As early as 2004, the symmetrical sulfonated urea salt, designated as arginine methylation inhibitor‐1 (AMI‐1), emerged as the inaugural antagonist of PRMT [209]. AMI‐1 has been identified as a potent antagonist of superoxide anions generated by NADPH oxidases, exhibiting superior antioxidant efficacy in comparison with established agents such as 4,5‐dihydroxy‐1,3‐benzenedisulfonic acid and 4‐hydroxy‐2,2,6,6‐tetramethylpiperidine‐1‐oxyl. Notably, the inhibitory action of AMI‐1 on NOX5 is exerted in a manner that is independent of intracellular calcium ion levels, underscoring its direct mechanism of action. Furthermore, the capacity of AMI‐1 extends to the suppression of superoxide anions produced by NOX1 and other NOX isoforms, while it notably lacks an effect on the activity of NOSs, including endothelial and inducible forms. This selectivity highlights the specificity of AMI‐1 for the NOX enzyme family [210]. AMI‐1 emerges as an efficacious inhibitor of PRMTs, attenuating the production of superoxide anions by NADPH oxidases through its intrinsic antioxidant mechanisms, rather than through modulation of enzymatic activity.

#### 6.1.2 MS023

MS023 stands out as a powerful, selective, and cell‐permeable inhibitor of human type I PRMTs. It displays remarkable selectivity, characterized by nanomolar IC50 values for multiple pivotal PRMT enzymes: 30 nM for PRMT1, 119 nM for PRMT3, 83 nM for PRMT4, 4 nM for PRMT6, and 5 nM for PRMT8. This specificity in inhibition highlights its potential as a therapeutic agent. The design and synthesis of MS023 were motivated by the structures of selective inhibitors for PRMT6 (EPZ020411) and PRMT4 (CMPD‐1). In formulating MS023, researchers preserved the ethylenediamine side chain present in the arginine mimetics of both EPZ020411 and CMPD‐1, which are considered the principal contributors to their inhibitory effects on PRMT4 and PRMT6, respectively. The pyrazole ring characteristic of EPZ020411 was strategically replaced with either a 1,2,3‐triazole or pyrrole ring to probe the influence on the electronic characteristics of the core heterocycle. This modification led to the conception and synthesis of MS023 and its congeners [211]. In the therapeutic landscape of acute lymphoblastic leukemia, MS023 exerts its potential through the inhibition of PRMT1 activity, leading to a diminution in the methylation of specific arginine residues R972/973 on the FLT3. This reduction in methylation subsequently disrupts the recruitment of adaptor proteins, a process integral to FLT3 signaling. Consequently, MS023 enhances the induction of apoptosis and curbs the proliferation of leukemia cells, thereby illustrating its promise as a therapeutic agent specifically for MLL‐rearranged ALL [212].

#### 6.1.3 ZJG51 and ZJG58

The pivotal role of PRMT1 in cervical cancer has established it as a promising therapeutic target. Using sophisticated molecular modeling, researchers have identified a novel sub‐binding pocket on PRMT1. This discovery has catalyzed the design and synthesis of a new class of compounds, featuring an innovative third substituent on the thiazole ring, crafted to specifically target and occupy this pocket with high affinity. Among these, ZJG51 and ZJG58 have demonstrated pronounced inhibitory effects on PRMT1 activity. ZJG51, in particular, has exhibited remarkable antiproliferative properties across a panel of cancer cell lines, including A549, MDA‐MB‐231, HepG2, and notably, HeLa, where it has shown exceptional potency with an IC50 value of 9.43 ± 0.10 µM. This underscores the profound therapeutic potential of ZJG51 as a candidate for the treatment of cervical cancer, suggesting a promising avenue for future clinical development [213].

#### 6.1.4 C7280948

C‐7280948 has been identified as a potent and selective inhibitor of human PRMT1, demonstrating antitumor activity through the specific suppression of its enzymatic function. The compound has underscored its capacity for optimization, as evidenced by structure–activity relationship analyses at the molecular level. These findings establish C‐7280948 as a potential lead chemical for the development of novel treatments targeting hormone‐dependent malignancies [214]. Research suggests that the PRMT1 inhibitor C‐7280948 may enhance the radiosensitivity of lung cancer by modulating the PRMT1/PKP2/β‐catenin/LIG4 signaling pathway, thereby overcoming radiation resistance and improving patient outcomes [99].

#### 6.1.5 TC‐E‐5003

TC‐E 5003 represents a highly selective inhibitor of PRMT1, exhibiting an IC50 value of 1.5 micromolar. It demonstrates the capacity to regulate the activation of the AP‐1 and NF‐κB signaling pathways induced by LPSs. As such, it possesses significant potential for further exploration and development as an anti‐inflammatory therapeutic agent [199]. TC‐E‐5003, a prospective antitumor agent, has demonstrated pronounced cytotoxic effects against a panel of cancer cell lines in vitro, including LC cells (A549 and NCL‐H1299) and BC cells (MCF‐7 and MDA‐MB‐231). The compound exhibited half‐maximal IC50 of 0.7022, 0.6844, 0.4128, and 0.5965 mM for the respective cell lines. Notably, the integration of TC‐E‐5003 with an innovative injectable in situ‐forming implant system (INEI) has been shown to markedly enhance tumor growth inhibition rates compared with the administration of TC‐E‐5003 in isolation. This advancement underscores the potential of INEI as a drug delivery platform to augment the therapeutic efficacy of TC‐E‐5003 [215].

#### 6.1.6 DCLX069

DCLX069 is recognized as a selective inhibitor of PRMT1, with an IC50 value of 17.9 µM, and shows diminished efficacy against PRMT4 and PRMT6 enzymes. An in vitro study has demonstrated that DCLX069, at concentrations between 12.5 and 100 µM, effectively inhibits the proliferation of cell lines indicative of BC (MCF7), liver cancer (HepG2), and AML (THP1) in a dose‐dependent manner. These results indicate that DCLX069 possesses significant antitumor potential, warranting further exploration as a therapeutic candidate [216]. Wang et al. [57] have elucidated the pivotal role of PRMT1 in the progression of GC. The targeted suppression of PRMT1 expression, achieved through the utilization of inhibitors such as AMI‐1 and DCLX069, significantly abrogated the metastatic and invasive properties of GC cells. This emphasizes the potential of PRMT1 as a viable therapeutic target in oncology, underscoring its involvement in the malignant evolution of GC [57].

#### 6.1.7 DCPT1061

Collaborative research by the Luo and Liu teams has revealed a novel function for PRMT1 in modulating CD8^+^ T cell infiltration and activation within the melanoma tumor microenvironment. The small‐molecule PRMT1 inhibitor DCPT1061 significantly increased the number and cytotoxic activity of tumor‐infiltrating CD8^+^ T cells, suppressed tumor growth, and sensitized treatment‐resistant melanomas to anti‐PD‐1 therapy [58]. In a separate line of investigation, the Liu team identified a novel mechanism of PRMT1 in BC pathogenesis. They developed a specific PRMT1 inhibitor, iPRMT1, which blocks the PRMT1‐mediated methylation of splicing factor SRSF1. This inhibition rectifies aberrant alternative splicing, thereby suppressing the inclusion of specific exons that drive BC cell proliferation. This discovery provides new avenues for developing PRMT1‐targeted antitumor drugs and for treating cancers driven by dysregulated RNA splicing [28].

#### 6.1.8 Furamidine Dihydrochloride (DB75 Dihydrochloride)

Furamidine dihydrochloride (DB75 dihydrochloride) has been characterized as a forceful, reversible, and selective inhibitor of both PRMT1 and TDP‐1. It exhibits marked selectivity for PRMT1, with an IC50 of 9.4 µM, and exerts a significantly diminished inhibitory effect on PRMT5, PRMT6, and PRMT4, corresponding to IC50 values of 166, 283, and above 400 µM, respectively. Furamidine dihydrochloride exerts its inhibitory action on both single‐stranded and double‐stranded DNA substrates, with a pronounced effect on the latter. Furthermore, it has been identified to possess antiparasitic activity, broadening its potential therapeutic applications [217].

### 6.2 Pan‐Inhibitor

#### 6.2.1 CTS‐2190

CTS‐2190 is a type I PRMT inhibitor that inhibits the activity of PRMT1 and its family enzymes, including PRMT3, PRMT4, PRMT6, and PRMT8. This inhibition impacts various biological processes in tumor cells, including epigenetic regulation, metabolic reprogramming, and DNA damage repair. This is the sole PRMT1 inhibitor currently identified in the clinical development stage according to available public information [68, 218]. A multicenter, open‐label phase I/II study (NCT06224387) sponsored by Cytosinlab Pharmaceutical Technology Co., Ltd. is assessing the safety, tolerability, pharmacokinetics, and preliminary antitumor efficacy of CTS‐2190 in patients with advanced or metastatic solid tumors. Part 1 involves a dose‐escalation phase, while Part 2 will expand cohorts in specific tumor types. Interim results show an objective response rate of 40% in a prescreened patient subset, suggesting promising antitumor activity in that population, although larger trials are needed for confirmation. Detailed adverse‐event data have not yet been disclosed, but the company states that the agent has a wide safety margin [20]. It was shown that combining this treatment with androgen receptor pathway inhibitors (e.g., enzalutamide), chemotherapeutic agents (e.g., docetaxel), or radiotherapy can improve antitumor efficacy, particularly in enzalutamide‐resistant metastatic castration‐resistant prostate cancer models [219].

#### 6.2.2 GSK3368715 (EPZ019997)

GSK3368715, or EPZ019997, is an orally active, reversible, noncompetitive inhibitor of PRMT1, with an IC50 of 3.1 nM. The inhibitor previously entered clinical trials but was subsequently discontinued due to dose‐limiting toxicities, specifically thromboembolic events, noted during the phase I trial. This indicates that targeting PRMT1 may inevitably disrupt normal physiological processes, raising safety concerns, resulting in the termination of clinical development. It has been shown to augment antitumor efficacy when administered alongside PRMT5 inhibitors, indicating a synergistic strategy in cancer therapy [220]. It can block PRMT1‐mediated methylation processes, therefore diminishing the proliferative activity of cancer cells. In vitro experiments have demonstrated the inhibitory effect of GSK3368715 on a lot of tumor cell lines, and it has also exhibited antitumor activity in animal models [221]. Furthermore, GSK3368715 has demonstrated strong sensitivity to ferroptosis in both in vitro and in vivo experiments for AML [222].

#### 6.2.3 II757

The design and synthesis of compound II757 were informed by the structure of the previously studied AH237, employing a propyl linker to conjugate thioadenosine with diversely substituted guanidino moieties. II757 demonstrates noncompetitive inhibition in relation to the peptide substrate H4‐21, while it exhibits pronounced competitive inhibition toward SAM, thereby characterizing II757 as a competitive antagonist for the SAM binding site [223]. Despite the existing dearth of comprehensive research on II757, its role as a pan‐PRMT inhibitor is underscored by its considerable inhibitory efficacy and selectivity. These attributes render II757 a robust and adaptable tool for the high‐throughput screening of PRMT inhibitors and a crucial building block in the assembly of novel PRMT inhibitors that exhibit enhanced cellular potency and refined selectivity profiles. RMT1 shows promise as a therapeutic intervention for various diseases. Nevertheless, there is a need for more selective and potent PRMT1 inhibitors. While some inhibitors have been developed, their selectivity and effectiveness need further improvement. Additionally, the specific mechanism of action and regulatory network of PRMT1 are not fully understood, which hinders its application in disease treatment. Thus, further research and development are required.

### 6.3 Challenges and Future Directions in the Development of PRMT1 Inhibitors

Current drug development efforts targeting PRMT1 face several significant challenges. The high degree of homology among PRMT family members, coupled with functional overlap and cross‐regulation, complicates the achievement of selectivity. For instance, inhibition of PRMT5 can induce a compensatory upregulation of PRMT1, suggesting that highly selective PRMT1 inhibitors or rationally designed dual PRMT1/PRMT5 inhibitors may be necessary for effective therapeutic outcomes. Furthermore, PRMT1 has a vast and diverse substrate repertoire, encompassing both histone and nonhistone proteins, and is involved in critical processes such as transcriptional regulation, RNA splicing, and signal transduction. This functional pleiotropy complicates the prediction of both the efficacy and the potential toxicity of PRMT1 inhibitors in vivo. Consequently, safety profiling is a paramount consideration for the clinical advancement of PRMT1 inhibitors. The thromboembolic events observed with GSK3368715 in clinical trials underscore the potential for serious mechanism‐based or off‐target toxicities. Therefore, a thorough evaluation of compound safety must be an integral component of all future PRMT1 inhibitor development.

The future development of PRMT1 inhibitors may involve combinations with other drugs, the creation of novel therapeutics, and the identification of new biomarkers. PRMT1 inhibitors are utilized alongside drugs with different mechanisms of action, including chemotherapeutic agents, targeted therapies, and immune checkpoint inhibitors, to enhance efficacy, mitigate drug resistance, and potentially lower the dosage of individual drugs to improve safety. Preclinical studies indicate that PRMT1 inhibitors, when used in conjunction with platinum‐based drugs, exhibit a synergistic effect in inhibiting tumor growth [218] or the HIF1α/P300 interaction inhibitor menadione [68]. In addition to traditional small molecule inhibitors, new technologies such as PROTACs are also being explored for targeting PRMT proteins, which may provide new therapeutic strategies [224]. The identification of biomarkers predicting the efficacy of PRMT1 inhibitors, including specific gene mutations, PRMT1 expression levels, or MTAP deletion status, can enable the precise selection of patient populations most likely to benefit from the therapy, thereby improving the success rate of clinical trials.

The clinical development of PRMT1 inhibitors remains in a preliminary phase. CTS‐2190 is presently the primary focus, and its development merits careful observation. Nonetheless, the challenges associated with GSK3368715 highlight the scientific difficulties in targeting PRMT1, particularly regarding safety concerns that require careful consideration. Future success will depend on a comprehensive understanding of PRMT1 biology, the identification of highly selective compounds, the development of rational combination strategies, and the discovery of biomarkers to inform patient selection.

## 7 Perspective and Conclusion

This review has synthesized the essential role of PRMT1 in diverse physiological and pathological contexts, highlighting its critical involvement in cancer, CLDs, CVDs, NDs, and immune‐related conditions. PRMT1 exerts its pleiotropic effects by catalyzing the methylation of a broad spectrum of histone and nonhistone substrates, thereby directly influencing gene expression, the DDR, cellular signal transduction, metabolic reprogramming, and immune regulation. Consequently, the dysregulation of PRMT1 is a common pathogenic driver in the onset and progression of numerous diseases. It functions as a potent oncogene, promoting tumorigenesis through unchecked proliferation, evasion of apoptosis, enhanced invasion and metastasis, and remodeling of the tumor microenvironment. In CLDs, PRMT1 is a key regulator of aberrant lipid metabolism, chronic inflammation, and fibrotic progression. Within the cardiovascular system, it critically modulates endothelial function, pathological myocardial hypertrophy, and the phenotypic switching of VSMCs. Similarly, PRMT1 influences neurodegenerative processes by modulating protein aggregation and neuronal survival, while in the immune system, it governs the activation and function of T cells, B cells, and macrophages. In summary, the evidence presented firmly establishes PRMT1 not merely as a significant regulatory molecule, but as a central node of disease pathogenesis and a compelling therapeutic target across a wide spectrum of human disorders.

Future research should focus on elucidating the mechanisms of PRMT1 across different diseases. The function of PRMT1 may vary significantly or exhibit opposing effects across different tissues, cell types, and disease stages. The observed variability necessitates the use of advanced models, such as conditional gene knockout and spatiotemporal‐specific regulation, for functional validation. PRMT1 exhibits various splice isoforms, each characterized by distinct enzymatic activities and substrate affinities. Their distinct expression and functional roles in diseases are not well understood, highlighting the necessity for additional research into the functional differentiation and regulatory mechanisms of each subtype.

PRMT1 has been identified as a potential target for therapeutic intervention. Small‐molecule inhibitors, including AMI‐1, MS023, TC‐E‐5003, C7280948, and the clinically staged CTS‐2190, along with emerging candidates such as DCPT1061 and ZJG51, have shown that PRMT1‐targeted therapy can inhibit disease progression, counteract resistance to PARP inhibition, chemotherapy, and radiotherapy, and enhance the efficacy of immune checkpoint blockade in various models of hematologic malignancies, BC, GC, HCC, melanoma, and immune‐related diseases. The discontinuation of GSK3368715 due to thrombotic events underscores that challenges related to inhibitor selectivity, toxicity, and drug resistance persist in clinical translation. Future research should prioritize the development of PRMT1 inhibitors that exhibit enhanced selectivity and reduced toxicity. Additionally, it is essential to investigate combination strategies with other treatment modalities, including chemotherapy, radiotherapy, and immunotherapy, to achieve synergistic effects and address resistance issues.

## Author Contributions

**Tao Wu and Lei Wang**: conceptualization, supervision, and writing – reviewing and editing. **Yanqun Luo**: data curation, methodology, software, and writing – original draft preparation. **Ying Gao and Xiaoliang Deng**: visualization and investigation. All authors have read and approved the final manuscript.

## Funding

This work was supported by the National Natural Science Foundation of China (82574670, 81973725), National Natural Science Foundation of Shanghai (24ZR1466600), and Excellent Project of 2024 Shanghai Oriental Talents (BJWS2024050).

## Ethics Statement

The authors have nothing to report.

## Conflicts of Interest

The authors declare no conflicts of interest.

## Data Availability

The authors have nothing to report.
